# Dynamic 3D chromatin organization and epigenetic regulation of gene expression in peanut nodules

**DOI:** 10.1111/jipb.70007

**Published:** 2025-08-13

**Authors:** Lixiang Wang, Chunhai Mai, Suqin He, Bingjie Niu, Gaiya Jia, Tao Yang, Yiwei Xu, Meng Ren, Xiaorui Zhao, Xin Liu, Zhaosheng Kong

**Affiliations:** ^1^ Shanxi Hou Ji laboratory, College of Agriculture Shanxi Agricultural University Jinzhong 030801 China; ^2^ Wuhan Frasergen Bioinformatics Co. Ltd. Wuhan 430075 China

**Keywords:** ATAC‐seq, gene expression, Hi‐C, nodule, peanut

## Abstract

Root nodules are specialized organs formed by the symbiotic relationship between legumes and soil‐borne rhizobia, facilitating an exchange of energy and nutrients essential for both organisms. This process is accompanied by dynamic changes in genomic organization and gene expression. While the three‐dimensional (3D) architecture of the genome is known to influence gene regulation, its role in nodulation and symbiotic nitrogen fixation remains largely unexplored. In this study, we present the first high‐resolution (40 kb) 3D genomic map of peanut roots and root nodules, generated using a high‐throughput/resolution chromosome conformation capture strategy. Compared to roots, ∼2.0% of chromosomal regions in nodules transition from a repressive (B) to an active (A) compartment and exhibit significant alterations in topologically associated domains (TADs). Peanut nodules also show more extensive *cis*‐interactions, with 100s of differentially expressed genes enriched in symbiotic pathways and nitrate metabolism. Additionally, assay for transposase‐accessible chromatin with high‐throughput sequencing identifies 25,863 and 14,703 open chromatin regions (OCRs) in roots and nodules, respectively. By integrating OCR mapping with epigenomic modifications, we reveal dynamic local OCRs (LoOCRs) and histone modifications associated with nodulation‐related genes. Notably, novel TADs and long‐range chromatin loops are detected in peanut nodules, including an H3K27me3 modification‐mediated loop that may regulate the expression of *Nodule Inception*. Another altered chromatin loop highlights the nodule highly expressed *AhMsrA* gene, which positively influences nodulation. Together, these findings shed new light on how chromatin architecture shapes gene expression during legume nodulation and nitrogen fixation.

## Graphical abstract

A 3D genomic map of peanut nodules revealed that the nodules exhibit chromatin reorganization, with 2% of regions transitioning to active states, altered topologically associating domains and enhanced cis interactions. The identification of chromatin loops that regulate nodulation genes links 3D genome dynamics to symbiotic nitrogen fixation.

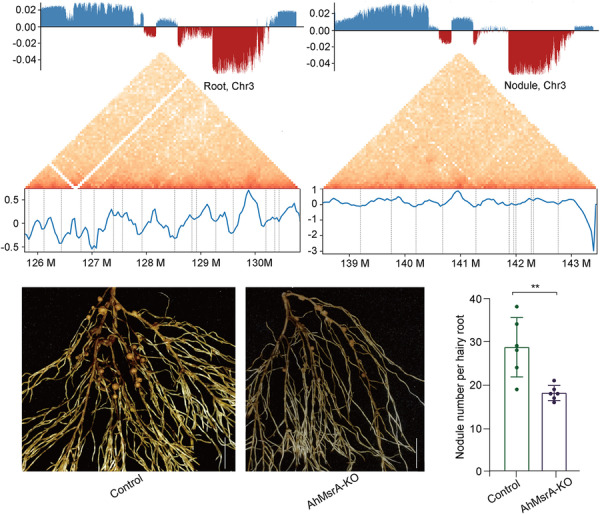

## INTRODUCTION

In eukaryotes, genomes are organized at different levels in the nucleus (Sotelo‐Silveira et al., 2018; Zheng and Xie, 2019). Chromatin folding, interaction, and genome organization play vital roles in gene expression regulation (Lieberman‐Aiden et al., 2009; Wang et al., 2015). Open chromatin regions (OCRs) in eukaryotic genomes are regulated by *trans*‐acting factors, including transcription factors (TFs), to modulate gene expression (Rodgers‐Melnick et al., 2016; Oka et al., 2017; Yan et al., 2019). High‐throughput chromosome conformation capture (3C)‐based methods, including high‐throughput/resolution 3C (Hi‐C) and chromatin interaction analysis, detect extensive local and distal *cis*‐regulatory elements (CREs) in various biological processes of mammals and plants. The three‐dimensional (3D) genome recruits the distant promoter, enhancer, and other CREs together with epigenetic marks, including DNA methylation, histone post‐translational modifications, and chromatin accessibility, to regulate gene expression (Chakraborty et al., 2023).

On the megabase scale, mammalian chromatin interaction patterns can be described as active euchromatic “A” and inactive heterochromatic “B” compartment regions; both can be converted to each other during cell differentiation. On the sub‐megabase scale, mammalian genomes can be partitioned into topologically associated domains (TADs) (Rodgers‐Melnick et al., 2016; Chakraborty et al., 2023), which are separated by boundary regions enriched for CCCTC‐binding factors (CTCF), cohesion, and specific epigenetic marks. TADs are largely conserved and may act as functional regulatory units for transcription regulation, chromatin states, and DNA replication (Berke et al., 2012; Beagan and Phillips‐Cremins, 2020). In plants, TADs have been characterized in maize (*Zea mays*), rice (*Oryza sativa*), soybean (*Glycine max*), and other plant species (Dong et al., 2017; Liu et al., 2017; Wang et al., 2021; Ni et al., 2023; Li et al., 2024). Chromatin loops between genes or between genes and distal elements have been widely detected during stress responses and organ development (Peng et al., 2019; Sun et al., 2020). However, how chromatin architecture reorganization shapes gene expression during legume nodulation remains largely unknown.

The legume–*Rhizobium* interaction leads to the formation of root nodules, involving the mass regulation of specific genes. Symbiotic nitrogen (N) fixation in legumes fixes 288 × 10^9^ atmospheric N into bio‐usable N each year for agriculture, and this interaction relies on root nodules formed with compatible rhizobium for N_2_‐fixing symbiosis (Yang et al., 2022a). Nodulation requires conserved mechanisms in legumes, including at least two chemistry and recognition systems. Rhizobia recognize plant‐excreted flavonoid signals, resulting in lipo‐chitin nod factor biosynthesis, and the bacterium nod factor is recognized by the plant host LysM family nod factor, followed by infection thread induction. The infection thread then extends into the root cortex region (inner cortex for indeterminate nodules and outer cortex for determinate nodules), and cortex cell division and expansion occur, resulting in the development of nodule premordia and then the final mature nodule (Oldroyd and Downie, 2008; Ferguson et al., 2019; Bozsoki et al., 2020; Ghantasala and Roy Choudhury, 2022). One mature root nodule is a multi‐species organ, consisting of 1,000s of plant cells and 6 × 10^9^ bacteroids; more specifically, nodules contain a nodule cortex, nodule endodermis, nodule parenchyma, vascular bundle, and infection zone (nodule meristem, especially for indeterminate nodules) (Luo et al., 2023). In nodules, a series of N fixation, N assimilation, and O redox genes are highly expressed, and each infected cell, called a symbiosome, carries out N fixation. Fixed N is transferred to the neighboring non‐infected cell. The outer cortex of the nodule and leghemoglobin consist of a physical barrier for O_2_ permeation, ensuring very low O_2_ levels in the nodule (Liu et al., 2023).

In addition to the more well‐studied root hair infection thread mode, 25% of legumes, including groundnut (*Arachis hypogaea* L.), use a unique rhizobium invasion strategy known as “crack entry,” which exists in a few legumes, including *Arachis*, *Sesbania*, and *Aeschynomene* (Quilbé et al., 2022). Except for genes shared in both modes, such as *NORK* (Nodulation Receptor Kinase), *NSP2* (Nodulation‐Signaling Pathway 2), *DMI3* (Doesn't Make Infections 3) and *Nodule Inception* (*NIN*), the molecular mechanism underlying the latter strategy is still unclear (Sinharoy and DasGupta, 2009; Peng et al., 2017, 2021). Based on a previous study, an important infection thread involving the “*RPG1* (Rhizobium‐Directed Polar Growth 1)” gene was lost in peanut (Griesmann et al., 2018). However, peanut NFR1 (Nod Factor Receptor 1) homolog gene knockout (KO) plants nodulate normally, suggesting that a novel phenomenon or even pathways may be involved in this infection mode (Shu et al., 2020).

Accessible chromatin regions (ACRs) and epigenetic modifications play crucial roles in gene expression across various biological processes, including nodulation. Studies on DNA accessibility and histone modifications have revealed correlations between ploidy levels and transcriptional reprogramming, illustrating the connections between chromatin changes and gene expression (Nagymihály et al., 2017; Wang et al., 2020). Differentially methylated regions (DMRs) are associated with nodule differentiation. Notably, reduced expression of demethylase DEMETER (DME) led to hypermethylation and subsequent defects in nodule formation. Additionally, significant dynamics in DNA methylation were observed during nodule development, particularly the CHH context. However, how 3D chromatin structure variations in legume nodules, and their associated ACRs, epigenetic modifications, regulate the expression of nodulation and nitrogen fixation‐relevant genes has not yet been reported.

In this study, the genome organization of peanut roots and root nodules were analyzed to uncover the 3D chromatin structure in nodulated peanuts. Using integrative analyses with Hi‐C, Whole Genome Bisulfite Sequencing (WGBS), transposase‐accessible chromatin with high‐throughput sequencing (ATAC‐seq) and RNA sequencing (RNA‐seq) data, we illustrated the genome architecture and expression regulation of nodulation‐involved genes in peanut. In this study, extensive local and distal OCRs and their epigenetic features were identified in peanut roots and nodules. A large number of chromatin loops were detected in the nodules and roots. The 3C experiment identified the different loop structures upstream of the first identified nodulation gene *NIN* (Schauser et al., 1999). Many nodule‐expressed genes, including some novel genes, were detected in altered loops between two tissues, and the accuracy of the analysis was validated in a functional study of the identified genes. Our results highlight how chromatin loops and epigenetic states regulate gene expression and identify novel nodulation genes from root and root nodule architecture.

## RESULTS

### Genome‐wide mapping of chromatin interactions in peanut roots and root nodules

To investigate chromatin organization in cultivated peanut nodules, high‐resolution Hi‐C maps were constructed using peanut roots and mature nodules (30 d after inoculation) through *in situ* Hi‐C analysis. After filtering the sequences, 2.81 billion pairs of sequencing reads (2.67 billion pairs of clean sequencing reads) were obtained from peanut tissues and mapped to the cultivated peanut reference genome (*Arachis hypogaea* cv. Tifrunner: assembly, annotation Version 1) (Table S1). We also conducted ATAC‐seq analysis (Table S2), three types of histone modification analysis (Table S3), DNA methylation analysis (Table S4), and RNA‐seq analysis (Table S5) using both the roots and nodules to determine how chromatin topology influenced transcriptional regulation during peanut nodulation. Nevertheless, the nodules contain a significant amount of bacterial DNA which may have resulted in the relatively low mapping rate of sequencing data of nodule samples; during the data analysis, we standardized the data to mitigate the effects of variations in data volume.

**Figure 1:**
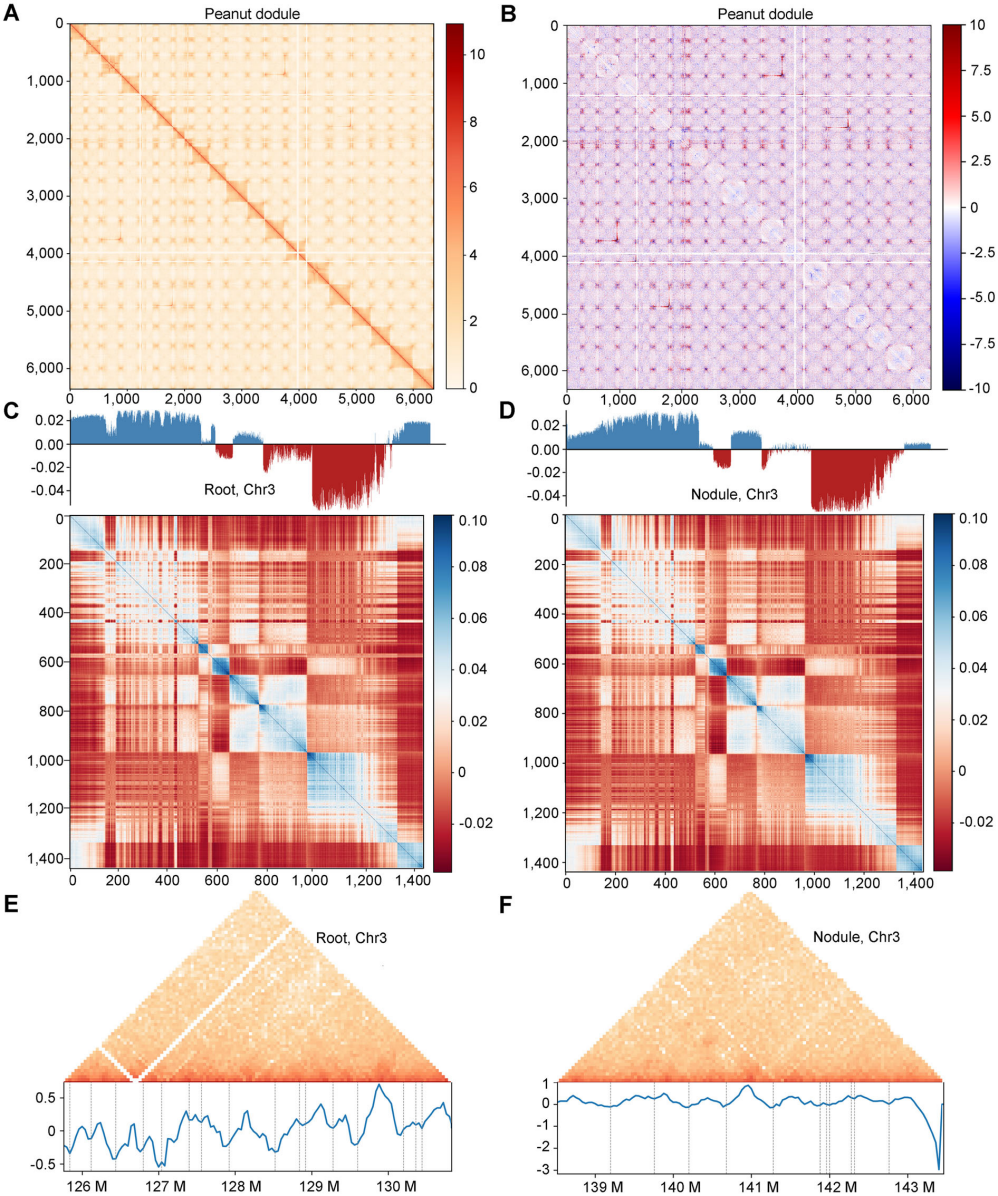
Hi‐C analysis of chromatin interactions in peanut roots and nodules **(A)** Chromatin interaction map represented by peanut nodules at a 400‐kb resolution. Color bars beside heatmaps indicate strong interactions in red and weak interactions in white. **(B)** Genome‐wide subtraction matrix of peanut roots versus nodules at a 400‐kb resolution. The interaction matrices of peanut roots and nodules were converted into *Z*‐score matrices; the *Z*‐score matrices of the two samples were subtracted to obtain the interaction subtraction matrix of these two samples. **(C**, **E)** Single chromosomal interactions in peanut roots at a 400‐kb resolution. The upper track shows Compartments A (blue histogram) and B (red histogram). The low track shows a chromatin interaction, represented by chr03 **(C)**. Each triangle distributed diagonally is represented as a topologically associated domain (TAD) in peanut roots **(E)**. **(D**, **F)** Single chromosomal interactions in peanut nodules at a 400‐kb resolution. The upper track shows Compartments A (blue histogram) and B (red histogram). The low track shows a chromatin interaction, represented by chr03 **(D)**. Each triangle distributed diagonally is represented as a TAD in peanut nodules **(F)**.

After confirming the reproducibility of biological replicates, we merged the replicates to increase the resolution of the Hi‐C matrix for the root and nodule samples. Comprehensive sequence analysis identified 908 and 829 million valid interaction pairs for the roots and nodules, respectively. These interactions were used to construct a 40‐kb resolution *in situ* Hi‐C map and a 400‐kb resolution subtraction matrix of peanut roots versus nodules (Figure 1A, B). We observed higher‐order chromatin structures, including compartments and TADs, on different length scales (Figure 1C–F; Tables S6, S7). We also generated simulated genome images of peanut roots and nodules (Figure S1).

### Chromosomal region interaction (Compartments A/B) in peanut roots and root nodules

Based on the single‐chromosome interaction matrix at 100‐kb resolution, we applied principal component analysis (PCA) to obtain the PC1 value of each bin and classified the peanut genome bins into two regions: “active” (Compartment A) and “inactive” (Compartment B). We conducted a compartment‐level comparative analysis to validate the conversion of Compartments A/B in different samples and the subsequent large‐scale changes in gene expression activity. At a 100‐kb resolution, three regions in peanut nodules (chr03, chr14, and chr15) had different distributions of Compartment A/B types compared with the roots (Figures 1C, D, S6). A total of 1,444 Compartment A and 1,413 Compartment B regions were detected in peanut roots, while 1,751 Compartment A and 1,791 Compartment B regions were found in peanut nodules. The compartment length of Compartment B was significantly longer than that of Compartment A in both tissues (Figure S3). The number of genes distributed in each bin of Compartment A was higher than that of Compartment B in peanut roots and nodules (Figure S4). A previous study has suggested that the Compartment A/B ratio may be associated with the genomic compartment (GC) content of genomic sequences (Fortin and Hansen, 2015). We observed that the GC content in each bin of Compartment B was higher than that in Compartment A in both peanut roots and nodules (Figure S4), consistent with the findings of a previous study (Rao et al., 2014).

**Figure 2:**
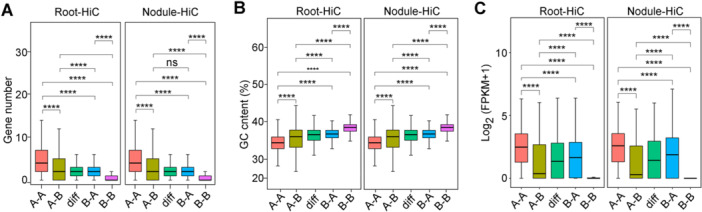
Genomic, ATAC, and epigenetic features of A/B compartments in peanut roots and nodules **(A**–**C)** Gene number **(A)**, genomic compartment (GC) content **(B)**, and gene expression (fragments per kilobase of exons per million mapped reads value) **(C)** of Compartment A, A to B switching compartments, different compartments, B to A switching compartments, and Compartment B, in peanut roots and nodules.

Compartments undergo A/B switching in different tissues and biological processes, resulting in significant gene expression changes (Barutcu et al., 2015). In this study, we identified 976 bins with A to B switching and 671 bins with B to A switching in the Nodule versus Root comparison across the entire genome. When compared with conserved A to A and B to B compartments, we observed an increased GC content in A to B compartments and higher gene density in B to A compartments. Conversely, B to A compartments showed a decreased GC content and lower gene density compared with A to B compartments (Figure 2A, B).

To determine the impact of compartment switching on peanut gene expression during nodulation, 1,647 bins were analyzed. From this analysis, 646 differentially expressed genes (DEGs) were identified with Compartment A/B switching in the Nodule versus Root comparison (Figure 2C). Gene Ontology (GO) analysis revealed that genes distributed in switched compartments were mainly enriched in cell, metabolic, and single‐organism processes. These genes were located in the cell, cell membrane, and cell organelle and functioned in molecular binding, catalytic activity, and transporter activity. Kyoto Encyclopedia of Genes and Genomes (KEGG) enrichment analysis further revealed that DEGs in A/B switching were involved in isoflavonoid biosynthesis, the calcium signaling pathway, and the carbon metabolism/carbon fixation pathway in prokaryotes, which play crucial roles in symbiotic N fixation. We also observed enrichment in peanut‐specific aflatoxin biosynthesis with nodule A/B switching, suggesting the potential regulation of peanut nodulation with aflatoxin biosynthesis (Figure S5).

### Mapping OCRs and epigenome marks in peanut roots and nodules

**Figure 3:**
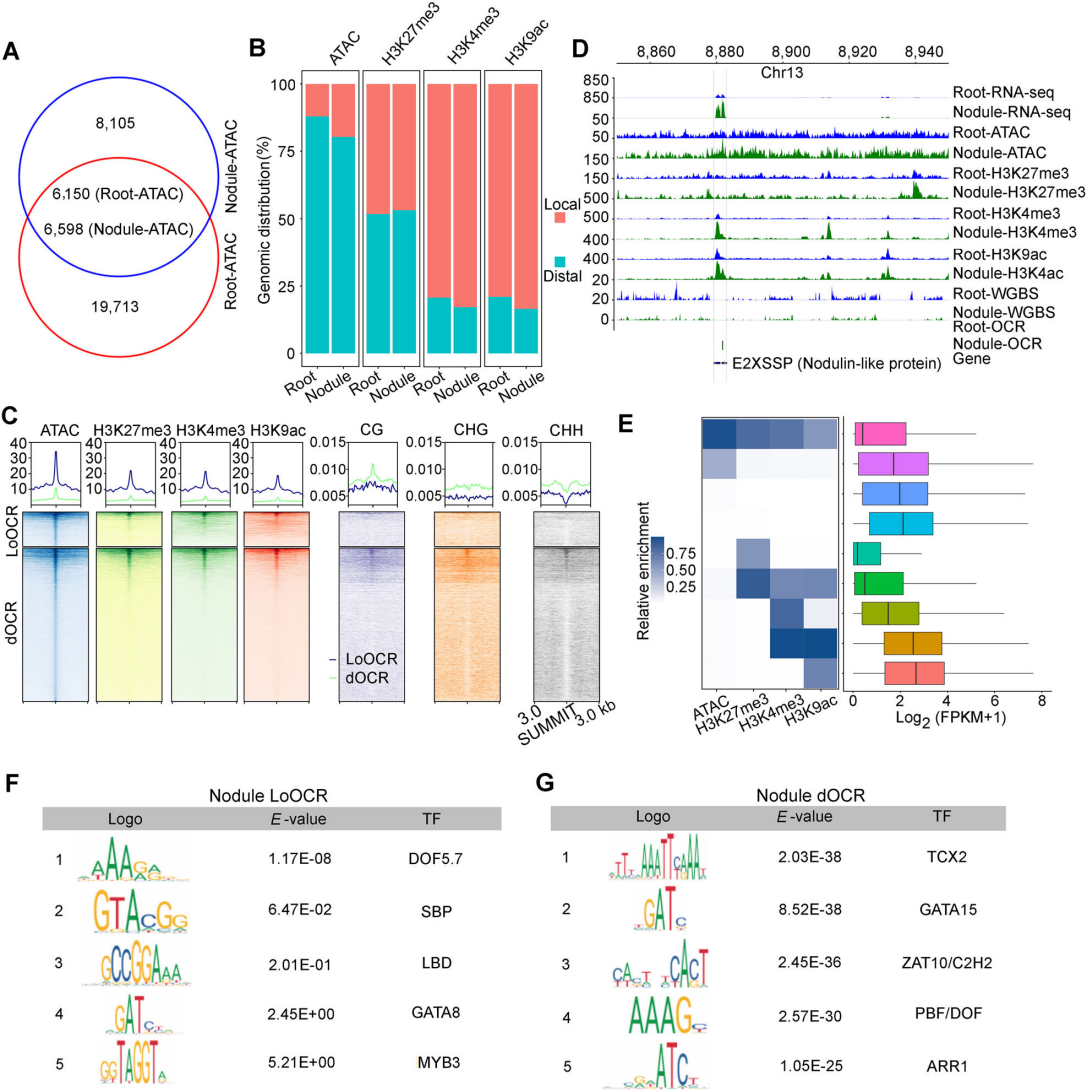
Characterization of OCRs and epigenome markers in peanut roots and nodules **(A)** Overlapped and tissue‐specific OCRs of nodules and roots. **(B)** Distribution of local and distal OCRs and three types of histone modifications (H3K4me3, H3K9ac, and H3K27me3) in roots and nodules. **(C)** Epigenome profiles of local OCRs (LoOCRs) and distal OCRs (dOCRs) centered on OCR summits in nodules. **(D)** Gene expression (RNA sequencing), chromatin accessibility (ATAC‐seq), H3K4me3, H3K9ac, H3K27me3, and DNA methylation (CG) profiles on a selected region of chromosome 13 (8,860‐8,940 kb). Vertical dotted lines show an example of dynamic chromatin accessibility and histone modifications with the altered transcription of a nearby gene between roots and nodules. **(E)** Correlation between gene expression and different clusters of chromatin accessibility and histone modifications in peanut nodules. **(F**, **G)** DNA motifs enriched in LoOCRs **(F)** and dOCRs **(G)** of nodules. The corresponding top five candidate motif‐binding transcription factors (TFs) are shown.

Open chromatin regions are regions in the eukaryotic genome that lack nucleosomes and typically contain CREs, such as promoters, enhancers, and insulators (Zhu et al., 2015). To investigate the CREs associated with DEGs during peanut nodulation, we used ATAC‐seq data to identify OCRs in both peanut roots and mature nodules. In two replicates, we detected 25,863 OCRs in roots and 14,703 in nodules, achieving highly reproducible results (Figure S6; Tables S2, S8). Of these, 19,713 OCRs were unique to roots, while 8,105 were unique to nodules (Figure 3A). This implies that nodules, as specialized N‐fixing organs, display distinct chromatin structures and gene expression patterns compared with peanut roots. Of the OCRs, 17.4% were situated near annotated genes and designated as local OCRs (LoOCRs). These LoOCRs extended from 3 kb upstream of the transcription start site (TSS) through the gene body to the TTS of the genes. The remaining OCRs (~82.6%) were outside of genic regions and categorized as distal OCRs (dOCRs), which may participate in long‐range chromatin interactions for transcriptional regulation (Table S9). To investigate the chromatin epigenetic features of flanking LoOCRs and dOCRs in roots and nodules, we generated global maps of three types of histone modifications (active H3K4me3 and H3K9ac and repressive H3K27me3) as well as WGBS DNA methylation (Tables S3, S4).

Analysis of ATAC‐seq and epigenome data sets demonstrated that LoOCRs in both roots and nodules exhibited higher levels of ATAC signaling and H3K4me3, H3K9ac, and H3K27me3 modifications than dOCRs, with nodules showing a higher local ATAC signal, H3K4me3 and H3K9ac modifications than roots (Figures 3B, C, E, S7), Nodulin‐like protein encoded gene *E2XSSP* was selected as an example to illustrate this (Figure 3D). Conversely, dOCRs displayed depleted levels of the ATAC signal and H3K4me3, H3K9ac, and H3K27me3 modifications (Figures 3C, S7). Moreover, the majority of LoOCRs and dOCRs showed low DNA methylation levels, although LoOCRs had even lower DNA methylation levels than dOCRs in nodules, suggesting distinct regulatory patterns. Genes with H3K4me3 and H3K9ac modifications exhibited significantly higher expression in this symbiotic organ (Figures 3C, E, S7). A correlation was established between gene expression and specific chromatin features compared with those with H3K27me3 modifications (Figures 3E, S7). In addition, the potential information of putative TF‐binding motifs enriched in LoOCRs and dOCRs was predicted. A significant enrichment of binding motifs for DOFs, IDD2, MYB, and LBD18 was observed in nodule LoOCRs and dOCRs. Nitrate‐responsive *cis*‐element (NRE) bound by NLPs and NIN are crucial for regulating the expression of nodulation genes. Our analysis identified 1,401 NREs containing root‐specific OCRs and 380 NREs containing nodule‐specific OCRs, with 68 and 22 genes associated with these root and nodule LoOCRs, respectively (Figures 3F, G, S7; Table S10). These findings indicate that OCRs utilizing CREs recruit diverse TFs to regulate gene expression.

### Dynamic LoOCRs and histone modifications associated with nodulation‐related genes

**Figure 4:**
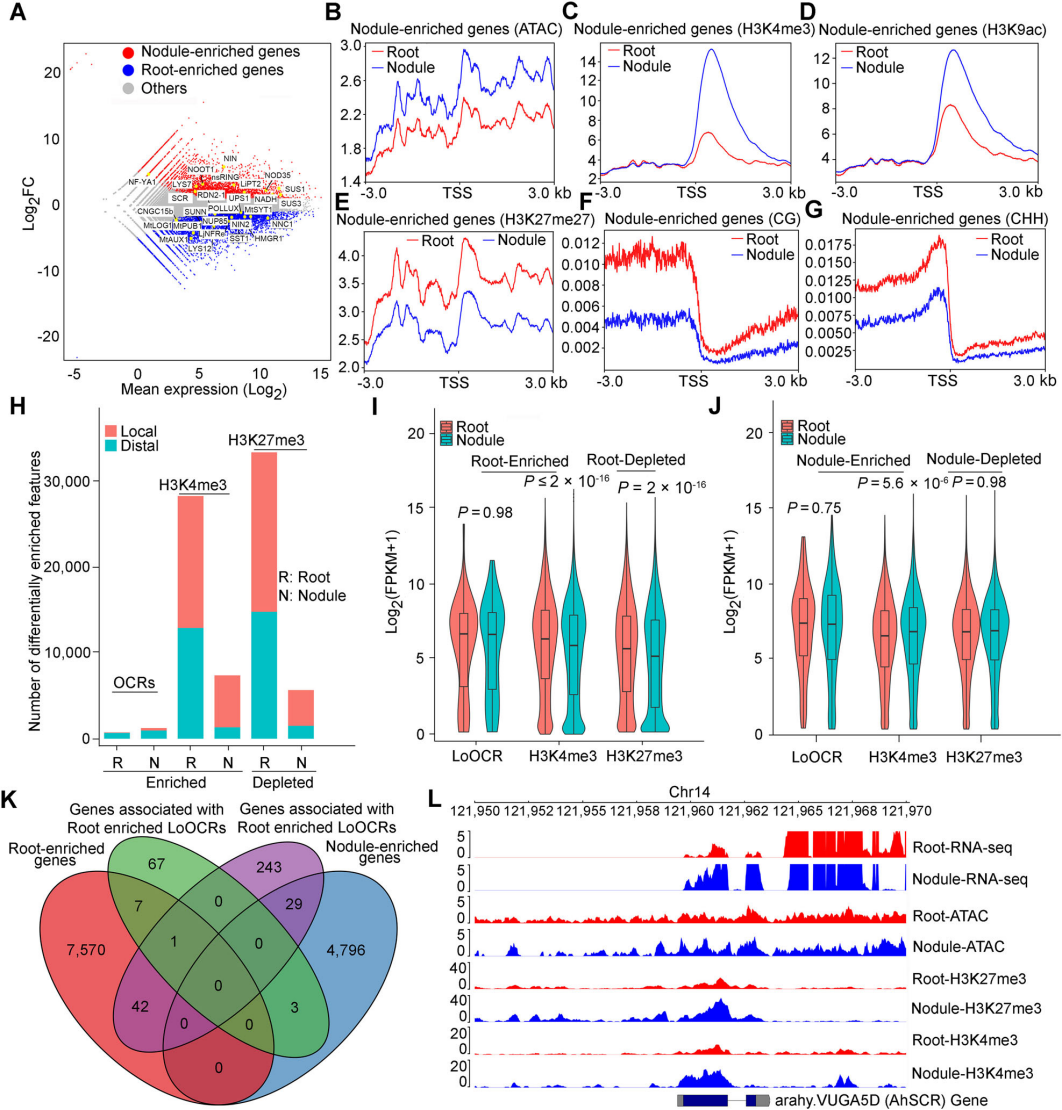
Dynamic LoOCRs and histone modifications associated with differences in gene expression between roots and nodules **(A)** MA (Log‐fold change and average expression) plot showing differentially expressed genes between roots and nodules, with some known nodulation‐related genes marked. Blue dots: 7,317 root‐enriched genes; red dots: 8,680 nodule‐enriched genes. Differentially expressed genes (DEGs) were defined with the following criteria: *q* value < 0.01, FC (fold change) >1.5. **(B**–**G)** Average signal intensities of transposase‐accessible chromatin with high‐throughput sequencing (ATAC‐seq) **(B)**, H3K4me3 **(C)**, H3K9ac **(D)**, H3K27me3 **(E)**, CG‐Whole Genome Bisulfite Sequencing (WGBS) **(F)**, and CHH**‐**WGBS **(G)** ± 3 kb around the transcription start sites (TSSs) of nodule‐enriched genes. **(H)** Number of differentially enriched local and distal OCRs and H3K4me3 and H3K27me3 modifications in roots and nodules. **(I**, **J)** Expression levels of genes with root‐enriched LoOCRs, H3K4me3, and root‐depleted H3K27me3 modifications in roots **(I)** and genes with nodule‐enriched LoOCRs, H3K4me3, and nodule‐depleted H3K27me3 modifications in nodules **(J)**. The Wilcoxon test was used to test significance. Ns: *P* > 0.05. **(K)** Venn diagram showing the overlap between tissue‐enriched genes and genes associated with tissue‐enriched LoOCRs. **(L)** Example of a nodule‐enriched gene, *SCR* (SCARECROW), showing differences in its expression and chromatin state in roots and nodules.

Transcriptional diversification in roots may be responsible for nodule formation in legumes. Our reproducible RNA‐seq data sets allowed us to identify 8,449 genes enriched in roots and 6,412 genes enriched in nodules (Figures 4A, S8; Table S5). In root nodules, significant expression of key genes involved in nodulation, such as *AhNINa*, *AhNSP2*, and *AhSUS1*, was observed (Kaló et al., 2005; Yan et al., 2019; Bhattacharjee et al., 2022; Yang et al., 2022b). Through GO and KEGG analyses, we determined that the DEGs between roots and nodules were enriched in pathways related to starch and sucrose metabolism, and plant hormone signal transduction (Figures S9, S10). Notably, nodule‐enriched genes were highly enriched in the N metabolism pathway, which plays a crucial role in nodule N fixation. In addition, our analysis of gene expression, chromatin accessibility, histone modifications and WGBS revealed that nodule‐enriched genes displayed increased chromatin accessibility, more H3K4me3 and H3K9ac modifications, fewer H3K27me3 modifications, CG and CHH methylation levels (Figure 4B–G).

Furthermore, we employed DiffBind to analyze the variations in OCR enrichment (including both local and distal OCRs) as well as H3K4me3 and H3K27me3 modifications between roots and nodules (Figure 4H; Table S11). Root‐enriched genes exhibited higher expression of H3K4me3 modifications, while root‐depleted genes showed higher expression of H3K27me3 modifications in roots than in nodules (*P* < 2.7 × 10^−9^, Wilcoxon test) (Figure 4I). Conversely, nodule‐enriched genes displayed higher expression of H3K4me3 modifications in nodules than in roots (*P* = 2.8 × 10^−4^, Wilcoxon test) (Figure 4J). There were more shared genes between root‐enriched LoOCRs and root‐enriched genes than nodule‐enriched LoOCRs and nodule‐enriched genes (Figures 4K, S11). We found 3,075 nodule‐enriched genes that correlated with changes in at least one type of epigenetic modification (Figure 4K; Table S11), including *AhNFYA1*, *AhNINa*, *AhNFRe*, *AhCNGC15b*, and *AhSCR* in peanut nodules (Figures 4L, S12) (Schauser et al., 1999; Lefebvre et al., 2010; Charpentier et al., 2016; Hossain et al., 2016; Magne et al., 2018; Murakami et al., 2018). Collectively, these results demonstrate the crucial role of OCRs and histone modifications in the transcriptional diversification of genes involved in peanut nodulation and N fixation.

### Long‐range chromatin loops in peanut roots and nodules

**Figure 5:**
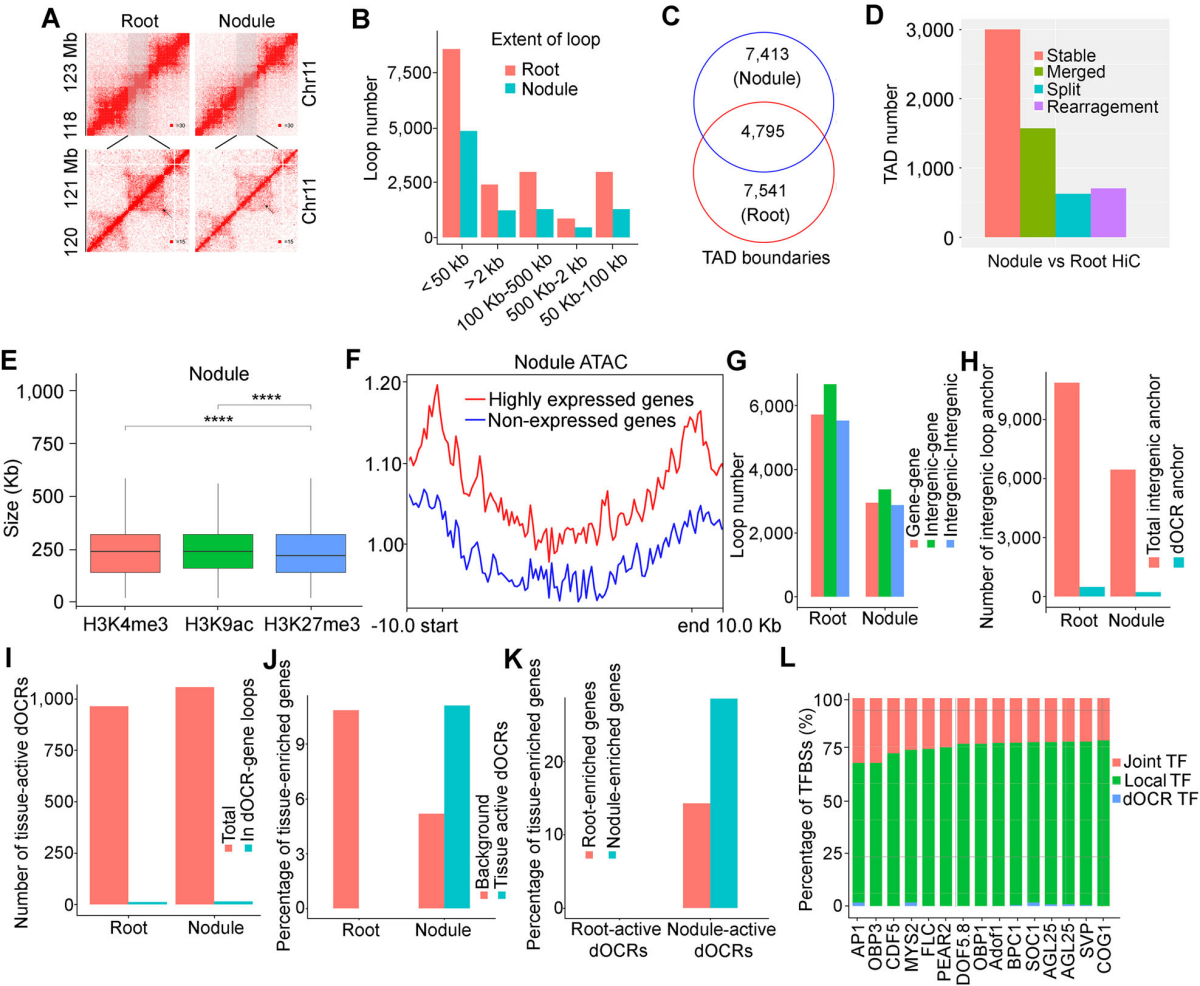
Characterization of TADs and loops and the dynamic activities of TF‐bound dOCRs **(A)** Chromatin interaction map of chromosome 11 in roots and nodules. **(B)** Number of chromatin loops with different interaction distances in roots and nodules. **(C)** Number of TAD boundaries and tissue‐specific TAD boundaries identified in roots and nodules. **(D)** Changes in TAD between roots and nodules. **(E)** Correlation between TAD size and different histone modifications in peanut nodules. **(F)** Highly expressed genes have higher chromatin accessibility together with their looped regions than non‐expressed genes in nodules. Highly expressed genes: fragments per kilobase of exons per million mapped reads (FPKM) > 100; non‐expressed genes: FPKM < 1. **(G)** Number of gene‐gene, intergenic‐gene, and intergenic‐intergenic loops detected in peanut roots and nodules. **(H)** Number of total intergenic anchors of loops that overlap with dOCRs in the root and nodule. Control: Same number of distal regions as dOCRs generated by randomly shifting dOCRs. **(I)** Number of tissue‐active dOCRs and tissue‐active dOCRs involved in dOCR–gene loops in roots and nodules. **(J)** Percentages of tissue**‐**enriched genes involved in tissue‐active dOCR–gene loops are significantly higher than those of tissue‐enriched genes involved in total dOCR–gene loops (background) in roots and nodules. **(K)** A comparison of the percentages of tissue‐enriched genes looped with tissue‐active dOCRs shows that root‐active dOCRs tend to interact with root‐enriched genes, while nodule‐active dOCRs tend to interact with nodule‐enriched genes. **(L)** Percentages of different types of TF‐binding sites (TFBSs) involved in dOCR–gene loops. dOCR TFBSs, TFBSs in only the dOCR anchors of dOCR–gene loops; Local TFBSs, TFBSs in only the gene anchors of dOCR–gene loops; Joint TFBSs, TFBSs on both anchors of dOCR–gene loops.

In peanut roots, we identified 51,122 interactions, with 17,914 *cis*‐interactions and 33,208 *trans*‐interactions. In peanut nodules, we found 33,768 interactions, with 9,194 *cis*‐interactions and 24,574 *trans*‐interactions (Figure 5A; Table S12). These extensive loops presented an opportunity to investigate the spatial relationships between CREs and target gene expression. Most chromatin loops spanned between 0 and 200 kb (Figure 5B), although we also observed some loops that were over 9,000 kb in length, indicating a potential mechanism for super‐distant gene regulation.

To further investigate the distinctions between roots and nodules at a high structural level, we conducted an analysis of TADs in peanut roots and nodules using a resolution of 40 kb. Initially, we did not observe significant variations in the relative TAD boundary signal values between roots and nodules. Subsequently, we compared the stability of the TAD boundaries and identified 4,795 overlapped boundaries, 7,541 boundaries specific to roots, and 7,413 boundaries specific to nodules (Figure 5C; Table S12). We then predicted the enriched putative TF‐binding motifs in the TAD boundaries of roots and nodules. The top five ranked enriching motifs in either the nodules or roots were WRKY binding sites, indicating that TAD boundaries recruit WRKY TFs to regulate gene expression (Table S13). A total of 4,386 genes (3,102 bins) were found in the TAD boundary of nodules, while 4,521 genes (3,080 bins) were distributed in the TAD boundary of roots (Table S14). KEGG enrichment analysis showed that the genes located in the TAD boundary of nodules were associated with N metabolism, lysine biosynthesis, photo‐transduction, and the plant circadian pathway (Figure S13). In addition, a comparison between roots and nodules revealed that peanut nodules had 1,573 merged, 628 split, and 706 rearranged TADs, indicating significant differences at the TAD level (Figure 5D). The insulation scale was used to measure the interaction between the two sides of each locus on the genome. In nodules, 297 genes located in 602 (0.95%) bins were identified in the insulation region compared with roots, and these genes were annotated by KEGG enrichment. Pathways such as endocytosis, zeatin biosynthesis, and proteasome were enriched in these genes (Figure S14).

The association of TAD boundaries with the gene expression level, chromatin accessibility, and epigenetic modifications was examined to identify genes that underwent significant structural changes during the nodulation process. Our findings revealed that TAD boundaries exhibited a notable enrichment of highly expressed genes, OCRs, and active epigenetic marks, such as H3K4me3 and H3K9ac. Conversely, there was lower enrichment of H3K27me3 in both root and nodule tissues (Figure 5E; Table S15). Notably, highly expressed genes showed higher chromatin accessibility than non‐expressed genes (*P* < 0.05, Wilcoxon test) (Figure 5F). We further classified the chromatin loops as gene–gene loops, intergenic–gene loops, and intergenic–intergenic loops (Figure 5G).

Genes located in nodule *cis*‐interaction were annotated to identify nodule‐specific loop‐containing genes. To identify peanut homolog genes, we used 332 reported nodulation‐related genes in *Medicago truncatula* as prey, resulting in 159 positive hits (Roy et al., 2020). Among these genes, 51 were located in the loop region (Table S16). A comparison between peanut roots and nodules revealed that some nodule TADs exhibited higher enrichment of H3K4me3 and lower H3K27me3 modifications, indicating a transition from inactive to active TADs during nodulation (Figure S15).

We visualized the Hi‐C matrix, Loop, ATAC, and chromatin immunoprecipitation (ChIP) of the loop regions near these 51 genes. *arahy.I65W25*, a homolog of *NIN* (the first identified TF involved in legume nodulation), was found to be highly expressed in peanut nodules. *AhNINa* was localized within a variable TAD of the root and nodule, enriched in H3K4me3 and H3K9ac modifications (chr13: 145,500,000–145,510,000; chr13: 145,530,000–145,540,000) (Figures 4A, S16). These results suggest that the delineation of the TAD and the associated epigenetic changes may be linked to peanut nodulation. In nodules, we identified a merged TAD with distinct chromatin loops compared to those in roots. Notably, one specific loop was absent in the nodules due to the presence of the H3K27me3 modification at the left border (chr13: 145,500,000–145,510,000) of the loop (Figure S16). We conducted 3C quantitative polymerase chain reaction (qPCR) experiments to validate the chromatin loops associated with *NIN* in both peanut roots and nodules (Figure S17; Table S17). The 3C‐qPCR results demonstrated that the relative frequency of chromatin loops was significantly higher in cross‐linked samples, underscoring the importance of chromatin architecture in regulating *NIN* expression, potentially nodulation and nodule nitrogen fixation.

### Identification of dOCR gene connections in peanut roots and nodules

The extensive dOCRs in peanut roots and nodules prompted us to investigate dOCRs that interact with their target genes through intergenic–gene loops. We found that only 4.1% (223/6,455 in nodule, 487/10,878 in root) of the intergenic anchors of all intergenic–gene loops contained at least one dOCR (Figure 5H). To investigate the impact of the dynamic activities of dOCRs on tissue‐specific gene expression, we focused on dOCRs that exhibited significant differences in accessibility or flanking repressive H3K27me3 modifications between roots and nodules (Table S18). These dynamic dOCRs were categorized as root‐active dOCRs (more open and/or weaker H3K27me3 in roots than nodules, *n* = 12) and nodule‐active dOCRs (more open and/or weaker H3K27me3 in nodules than roots, *n* = 15). Our findings revealed that 1.2% of root‐ (12/964) and 1.5% of nodule‐ (15/1,057) active dOCRs were involved in dOCR gene loops in roots and nodules, respectively.

The nodule dOCR–gene loops revealed there may be an interaction between *NRT1.1* (LZ85EL), nodulin MtN21 (L4R73E), *ERN1* (J0DG7J), *MATE1* (HSA1AU), sulfite exporter (FYY1I0), *ankyrin* (4YMK55), *syntaxin* (IINC9K), and their long‐distance regulatory regions (Figure 5I; Table S19). Furthermore, these nodule‐active dOCRs tended to interact with nodule‐enriched genes (Figure 5J, K; Table S20). These findings suggest that changes in gene expression between roots and nodules are influenced by dOCR dynamics. To further investigate this, we analyzed TF‐binding events at OCRs within the 3D genome context. Interestingly, we observed that 4%–30% of TF‐binding sites involved in dOCR–gene loops were found at both anchors of the loops, while the remaining TF‐binding sites were located either at the dOCRs' anchor or at the gene anchor (Figure 5L). This indicates that the transcriptional regulation of genes requires the collaboration of distal and local OCRs in recruiting relevant gene expression regulators.

### Identification of nodule‐specific loop genes and functional analysis of the *AhMsrA* gene in peanut nodulation

**Figure 6:**
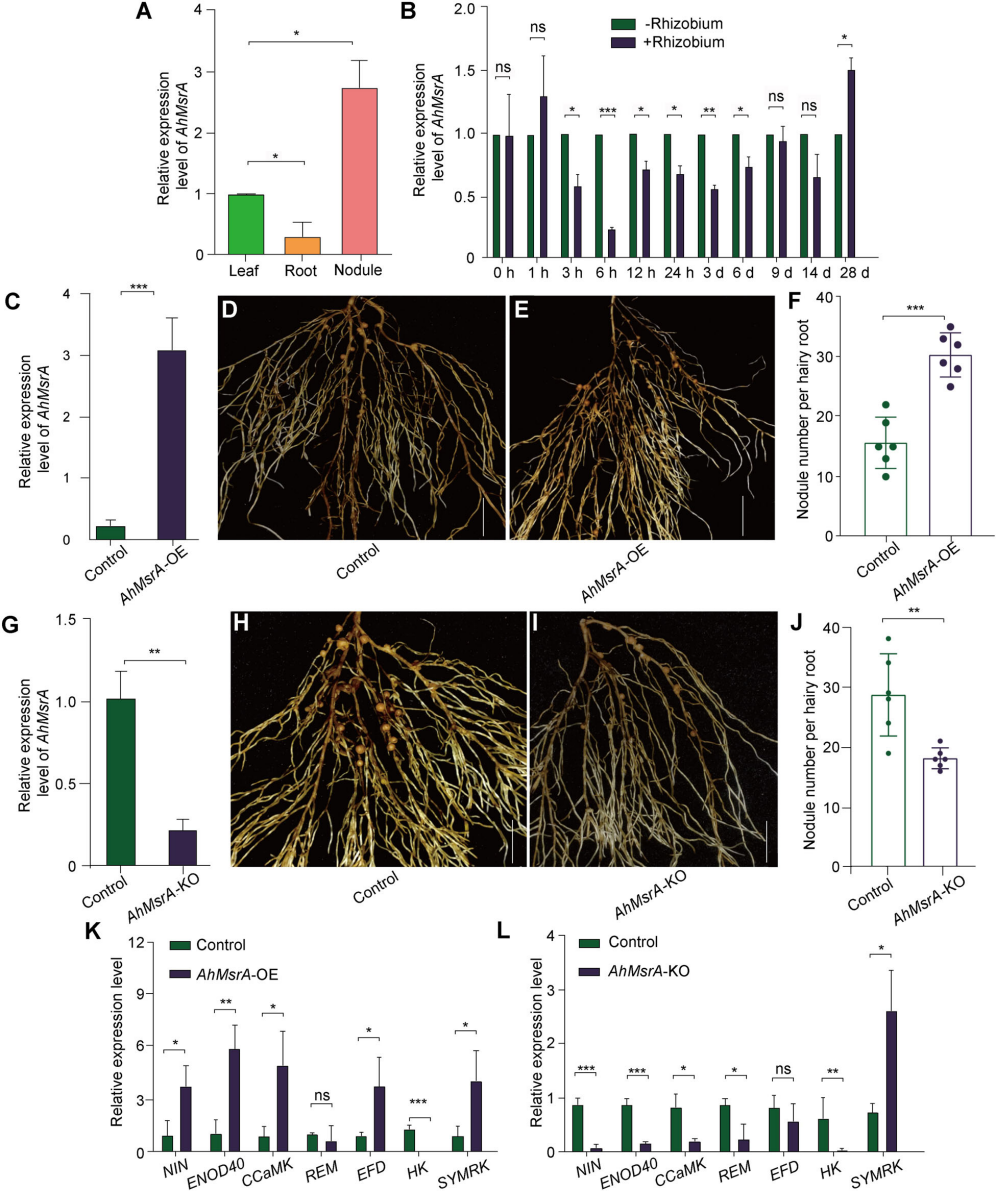
**Expression pattern and function of** ***AhMsrA*** **during peanut nodulation** **(A**, **B)** Relative *AhMsrA* expression in peanut leaves, roots, and nodules **(A)** and at different stages (0, 1, 3, 6, 12, and 24 h after inoculation (HAI); 3, 6, 9, 14, and 28 d after inoculation (DAI)) **(B). (C**–**F)** Phenotypic analysis of *AhMsrA* overexpression during peanut nodulation. Expression level of transgenic hairy roots harboring the empty vector and *35S:AhMsrA* **(C)**. Expression levels were normalized against the housekeeping gene of *AhActin*. Student's *t*‐test was performed (***P < 0.001, *n* = 15). Nodulation of transgenic roots expressing empty vector as control **(D)** and *35S:AhMsrA* **(E)** at 28 DAI. Bar = 1 cm. Quantitative statistics of nodule number per hairy root carrying EV and *35S:AhMsrA* at 28 DAI. Values are the mean ± *SD*. For each biological replicate, 12 hairy roots were collected (*n* = 6, Student's *t*‐test; ***P < 0.001) **(F). (G**–**J)** Phenotypic analysis of *AhMsrA* knockout (KO) during peanut nodulation. Expression level of transgenic hairy roots harboring empty vectors and *AhMsrA*‐KO (knock out). The expression levels were normalized against the housekeeping gene of peanut *AhActin*. Student's *t*‐test was performed (***P < 0.001, *n* = 15) **(G)**. Nodulation of transge*n*ic roots expressing empty vector as control **(H)** and *AhMsrA*‐KO **(I)** at 28 DAI. Bar = 1 cm. Quantitative statistics of nodule number per hairy root carrying EV and *AhMsrA*‐KO at 28 DAI. Values are the mean ± *SD*. For each biological replicate, 12 hairy roots were collected (*n* = 12, Student's *t*‐test; ***P < 0.001) **(J). (K**, **L)** *AhMsrA* positively regulates the expression of peanut symbiotic marker genes. Relative expression of nodule‐associated marker genes (*AhCCamK*, *AhHK1*, *AhNIN*, *AhSymREM1*, *AhEFD*, and *AhENOD40*) in *AhMsrA*‐OE (overexpression) roots compared with the control at 28 DAI **(K)**. Expression of nodule‐associated genes in *AhMsrA*‐KD (knockdown) roots compared with vector control roots at 28 DAI **(L)**. The transcript levels of *AhCCamK*, *AhHK1*, *AhNIN*, *AhSymREM1*, *AhEFD*, and *AhENOD40* were set to “1” in 28 DAI empty vector control roots. The expression of these genes was normalized to housekeeping gene *AhActin*. All values are the mean ± *SD* of three independent experiments (*P < 0.05, **P < 0.01, ***P < 0.001; *n* = 10).

There may be new genes involved in peanut nodulation, as peanut rhizobia use a “crack entry” strategy to infect the host. To identify these new genes, we conducted a joint analysis using ATAC and transcriptome data. We detected 1,277 DEGs located in 1,212 nodule‐specific loops as well as 495 nodule upregulated genes located in 504 loops (Tables S21, S22). By cross‐referencing the expression of these genes upregulated in nodules with the transcriptomes of 29 peanut tissues, we identified 71 putative nodule‐specific genes. One example is the *arahy.4A36F8* gene, which was located in a *cis*‐interaction region (chr13: 1,880,000–1,940,000) and specifically expressed in nodules. Our findings indicate that peanut nodules exhibit higher levels of H3K4me3 and H3K9ac modifications compared to roots, while peanut roots show a greater prevalence of CG and CHG type methylation than nodules. A significant enrichment of ATAC signals can be detected in the loop region of the *AhMsrA* gene, as opposed to the gene body region. Furthermore, various nodule CRE motifs, including DOF, MYB, TCX, ZAT, and ARR, are present in the promoter region of the *AhMsrA* gene (Figures 6A, S18).

*AhMsrA* significantly responded to rhizobium inoculation at almost all checkpoints (Figure 6B). This gene encodes a peptide methionine sulfoxide reductase called *AhMsrA*. However, the function of the homologous gene in the infection strategy of the infection thread has not yet been studied in legumes. We queried whether *AhMsrA* plays a role in peanut nodulation. To address this, we performed a systemic phenotypic analysis of *AhMsrA* using the hairy root transformation system to overexpress or knock out (KO) this gene in roots. Initially, a construct containing the CaMV35S promoter (35S): *AhMsrA* was transformed to obtain *AhMsrA*‐overexpressing transformed roots (*AhMsrA*‐OE). The nodulation status of the transformed *AhMsrA*‐OE roots was evaluated using enhanced green fluorescent protein (eGFP) fluorescence and 28 days after inoculation (DAI) using *Bradyrhizobium* (B.) *japonicum* CCBAU051107. Interestingly, the *AhMsrA*‐OE roots produced significantly more nodules compared with the control roots (Figure 6C–F). The average number of nodules per vector control root was 16.2, whereas the average number of nodules per *AhMsrA*‐OE root was 30.7. Thus, the number of nodules per *AhMsrA*‐OE root increased by ∼89.5%. To investigate the role of endogenous *AhMsrA* in peanut nodulation, *AhMsrA‐*RNAi was first applied to knockdown the internal *AhMsrA* messenger RNA (mRNA), as shown in Figure S19 of Supporting Information. The number of nodules was significantly reduced in *AhMsrA‐*RNAi roots compared with the vector control. The average number of nodules per root was 31.2 in the vector control but 15.7 in *AhMsrA‐*RNAi root. Thus, the total number of nodules per root increased by ∼49.6% in *AhMsrA‐*RNAi. We then generated CRISPR‐Cas9‐*AhMsrA* construct to disrupt the *AhMsrA* gene and examined nodulation in *AhMsrA*‐KO mutant roots (Figure 6G–J). The transformed *AhMsrA*‐KO roots were identified using eGFP fluorescence and single root sequencing (Figure S20). The CRISPR‐Cas9‐*AhMsrA* transgenic roots with sequence alterations exhibited a significantly reduced number of nodules compared with the vector control (Figure 6G–J). The average number of nodules per root was 28.2 in the vector control but 17.1 in the *AhMsrA*‐KO mutant. Consequently, the total number of nodules per root decreased by ∼39.3% in the *AhMsrA*‐KO mutant. These findings indicate that *AhMsrA* plays a positive role in regulating nodulation in peanut.

### Characterization of peanut nodulation‐relevant enhancers and their associated genes

Dynamic enhancer landscapes play a role in cell differentiation and organ development processes in a tissue‐specific manner, influencing target gene transcription in mammals and plants (Li and Leonard, 2018; Andersson and Sandelin, 2020). We conducted an analysis of three types of histone modifications and identified four distinct cases. Case 1 corresponded to a strong H3K27me3 signal, and Case 2 included all modifications with weak signals. Case 3 was characterized by a strong H3K4me3 and H3K9ac signal, and Case 4 exhibited a strong H3K9ac signal. Considering the regulatory element signals associated with these histone modifications, H3K4me3 and H3K9ac signals were selected as relatively active enhancers/promoters (Figure S21). In roots, we detected 2,710 putative enhancers by removing the promoter region (3 kb upstream and downstream of TSS), while in nodules, we detected 4,533 putative enhancers. By comparing the two, we found that peanut nodules contained 3,921 increased and seven decreased H3K4me3 enhancers as well as 1,380 increased and 28 decreased H3K9ac enhancers (Tables S23, S24).

To investigate the associated genes of the differential enhancer region, we analyzed the overlap between the differential enhancer regions and the loop anchors of each sample. Enhancers with the overlap anchor were used to analyze the overlap between the other end of the anchor and the promoter region of the gene. We screened 783 root‐specific and 266 nodule‐specific genes (with 317 shared genes in both samples) that had gain enhancers, while no genes interacting with loss enhancers were identified (Table S25). Gene Ontology analysis showed that the genes linked by root gain enhancers were enriched in starch metabolic process, cellulose synthesis activity, glucan metabolic process, glucan synthesis process, cellular polysaccharide, and carbohydrate metabolic terms. KEGG pathway analysis revealed that these genes were enriched in isoflavonoid biosynthesis and calcium signaling pathways. The genes associated with nodule gain enhancers were overrepresented in several metabolic processes, including protein modification, lipid glycosylation, hemicellulose metabolic process, and cell wall biogenesis. KEGG pathway analysis revealed that these genes were enriched in SNARE interactions in vesicular transport and isoflavonoid biosynthesis pathways (Figure S22). Finally, we conducted association study of the nodule‐enriched enhancers with representative nodulations genes and nodule‐specific CREs; two nodulation genes *98W9NM* (ARR) and *N099DK* (Legumain), two nodule‐specific OCRs (chr7: 67,947,404‐67,947,804; chr15: 154,340,629‐154,341,029) containing ARF, BPC, AS2/LOB, MYB, RAP/ERF, GATA and bZIP motifs were found to be associated with enhancers that are enriched in nodules (Figure S23).

## DISCUSSION

Legume symbiotic N fixation (SNF) contributes nearly half of the N provided by chemical fertilizer N (Canfield et al., 2010). Nodulation and SNF are critical agronomic traits for the ecosystem and legumes (Ferguson et al., 2019). Understanding the molecular mechanisms related to nodule initiation, development, and SNF can provide a genetic basis for improving legume yield and sustainable N source production. Forward and reverse genetics play a vital role in identifying the genes involved in these processes (Roy et al., 2020). Nearly 200 legume genes required for effective symbiosis have been identified to unravel the complexity of this process. Recently, epigenetic modifications, such as DNA methylation, histone modification, and small RNAs, along with functional CREs, have been found to play significant roles in fine‐tuning gene transcription in various biological processes. During legume nodulation, the roles of a growing number of microRNAs (miRNAs) that function during legume nodulation have been studied (Hoang et al., 2020). DEMETER (MtDME), a demethylase, is essential for nodule development and to regulate the expression of genes related to nodule development and differentiation (Nguyen et al., 2023). (NCR) (Nodule cysteine‐rich) peptides genes showed 44% hypomethylation and 4% hypermethylation, and ATAC‐seq analyses showed that highly expressed NCR genes, along with the highly enriched active histone mark H3K9ac and the less enriched repressive histone mark H3K27me3, had high DNA accessibility (Satgé et al., 2016; Yang et al., 2025). In this study, nodulation CREs were first identified using ATAC‐seq and epigenomic modifications in peanut root nodules. By integrating public nodulation‐related genes and peanut nodule‐enriched genes, we provide valuable information for understanding the regulation of nodulation and SNF, such as *AhNFYA‐10*, *AhNINa*, *AhNSP2*, *AhNOOT1*, *AhUricase‐2* (Nguyen et al., 2023), and *AhGS2* (Wang et al., 2013). We found that many highly expressed genes in nodules were associated with ATAC signaling and epigenetic modifications. Most nodule‐specific expressed genes presented combined epigenetic modification changes (Figures 4, S12). Based on our findings, DEGs related to peanut nodulation were closely associated with chromatin accessibility and epigenetic modifications. However, our association analysis revealed that the ATAC signals and histone modifications of root‐enriched genes are lower than those in nodules. In contrast, methylation modifications are found to be higher in nodules compared to roots. This suggests that the repressive H3K27me3 modification may play a significant role in gene diversification in peanut roots and nodules, which requires further investigation for clarification.

The genome was tightly packed for efficient storage of genetic information in limited nuclei. Histones compact DNA into the microscopic space of the eukaryotic nucleus, resulting in a DNA–protein complex called chromatin. The 3D organization of chromatin regulates many genome functions (Zheng and Xie, 2019; Chakraborty et al., 2023). Recently, Hi‐C sequencing has become an economical method for generating chromosome‐scale scaffolds, which can assist in defining the spatial organization of chromatin. Plant genomes contain mammalian‐like A/B compartments and extensive chromatin loops. The Hi‐C interaction matrix can partition chromosomes into A/B compartments and TAD domains (Lieberman‐Aiden et al., 2009). In this study, we identified 1,751 Compartment A and 1,791 Compartment B regions in peanut nodules, with 976 bins with A to B switching and 671 bins with B to A switching in the Nodule versus Root comparison across the entire genome. In addition, 3,102 boundaries specific to the nodule were characterized with 1,573 merged, 628 split, and 706 rearranged TADs. Our findings revealed that TAD boundaries were notably enriched in highly expressed genes, OCRs, and active epigenetic marks, such as H3K4me3 and H3K9ac. Chromatin structural changes in peanut roots and nodules suggest that the DEGs identified between peanut roots and nodules were involved in different biological processes. Several motifs around the TAD boundaries were found in peanut nodules, and further studies should explore the putative roles of motifs in TAD organization in peanut nodules.

Extensive rearrangements in the *Drosophila* genome have been reported to cause many changes to chromatin topology, disrupting long‐range loops and TADs (Ghavi‐Helm et al., 2019). The combination of Hi‐C, RNA‐seq, and ATAC‐seq data is beneficial for determining the correlation between chromosome conformation and gene regulation. Many recently published Hi‐C data sets represent a valuable resource for future profound understanding of the evolution of 3D chromosome organization in plants. A chromatin loop at WUSCHEL (WUS) has been shown to repress *WUS* expression *in planta* during flower development in Arabidopsis (Guo et al., 2018). However, the superior quality of the legume genome assembly highlighted the expansion of gene families contributing to legume common trait nodulation or one legume‐specific feature, such as gravitropism in groundnut and drought tolerance in chickpea (Garg et al., 2022). During legume nodulation, a remote upstream *cis*‐regulatory region was required for *NIN* expression in the pericycle, and nodule organogenesis suggests that it may present a chromatin loop in this process (Liu et al., 2019). We found that a chromatin loop (chr13: 145,533,068–145,536,074) at *NIN* was lost in peanut nodules (Figures 4A, S15, S16). Further study of the loop connections and the transcription factors of *NIN* will provide direct evidence for these data. In addition to known genes, we identified 100s of putative peanut nodulation genes in the nodule‐specific chromatin loops. We selected one of these genes, named *AhMsrA*, to validate the availability of these candidate genes. *AhMsrA* was mainly expressed in peanut nodules, significantly responded to peanut rhizobial infection, and positively regulated peanut nodulation (Figure 6). In summary, our comprehensive epigenomic analysis, high‐resolution 3D genome maps, and distal and local OCRs offer a profound understanding of peanut nodulation. We provided valuable annotations of the underlying *cis*‐ and *trans*‐regulatory mechanisms that contribute to DEGs in nodules. These findings provide valuable insights into the mechanisms of legume nodulation and offer potential strategies for improving N utilization efficiency.

## MATERIALS AND METHODS

### Plant materials and growth conditions

Peanut accession Nongdahua 103, kindly provided by professor Dongmei Yin from Henan Agriculture University, was used to conduct the project. For root samples, peanut plants were grown in sterile vermiculite in a growth room at 26°C under short‐day conditions (12‐/12‐h light/dark, fluorescent light; 200 L/mol/m^2^/s light intensity). For nodule samples, peanut plants were inoculated with *Bradyrhizobium arachidis* CCBAU051107 under the same growth conditions (optical density at 600 nm = 0.08). Peanuts were supplied with a low‐N nutrient solution containing 500 μmol/L KNO_3_, 680 μmol/L CaCl_2_, 490 μmol/L MgSO_4_, 730 μmol/L KH_2_PO_4_, 420 μmol/L Na_2_HPO_4_·12H_2_O, 80 μmol/L Fe‐Na‐ethylenediaminetetraacetic acid, 1500 μmol/L KCl, 46 μmol/L H_3_BO_3_, 10 μmol/L MnSO_4_·H_2_O, 0.77 μmol/L ZnSO_4_·7H_2_O, 0.32 μmol/L CuSO_4_·5H_2_O, and 0.54 μmol/L Na_2_MoO_4_·2H_2_O. Peanut roots (aged 1 week) were inoculated with rhizobia, and nodules were collected at 30 DAI. *Bradyrhizobium arachidis* strain CCBAU051107 was cultured for 2 d at 28°C in Yeast Extract Mannitol Agar medium. Two independent pools of roots and nodules were collected as two biological replicates for library construction of Hi‐C, ATAC‐seq, ChIP sequencing (ChIP‐seq), MethylC‐seq (one replicate), and RNA‐seq (three replicates).

### Chromatin immunoprecipitation sequencing analysis

After filtration of the interference information using Trimmomatic (Bolger et al., 2014) and quality assessment using FastQC (Andrews, 2014) in paired‐end raw data, we obtained clean data through quality control. Bowtie2 was then used to map clean reads to the reference genome (Langmead and Salzberg, 2012) (https://www.ncbi.nlm.nih.gov/assembly/GCA_003086295.2), and Samtools (Li et al., 2009) was applied to screen out the low‐quality mapped reads. Polymerase chain reaction redundancy, organelle alignment (v1.9, with default parameters), and Picard (with default parameters) were used to obtain the retained valid pairs for subsequent analysis. We used MACS2 (Feng et al., 2012) (v2.1.1.20160309) software based on statistical methods to perform peak calling (with parameters: ‐f BAMPE ‐B‐‐SPMR‐‐keep‐dup all), where the peaks called were significantly enriched regions from retained valid pair data for sequencing experiments with biological replication. Subsequently, R scripts were run with Deeptools software (Ramírez et al., 2016) for signal enrichment analysis of peak regions, gene body regions, and regions near the TSS using default parameters. We also used ChIPseeker (Guo et al., 2018) to obtain gene annotations on peak regions at the same time. Finally, we randomly selected the enrichment regions on the genome and visualized the called peaks of all samples using the Integrative Genomic Viewer (IGV) (Garg et al., 2022).

### Transposase‐accessible chromatin with high‐throughput sequencing analysis

Prior to mapping, standard next‐generation sequencing quality control steps using cutadapt (1.9.1) as well as ATAC‐seq‐specific quality control steps were performed. Trimmed reads were aligned to reference genomes using Bowtie2 (version 2.2.6) (Langmead and Salzberg, 2012), and reads mapped to the mitochondrial genome were removed using removeChrom (https://github.com/jsh58/harvard). Polymerase chain reaction duplicates were removed, and the number of mapped reads downsampled to a standard of ~20 mol/L were identified in the samples using Picard (version 1.126) (http://broadinstitute.github.io/picard/). We checked the insert size distribution of sequenced fragments to evaluate the ATAC‐seq data. When the insert size distribution had a clear periodicity of ∼200 bp, it suggested that many fragments were protected by integer multiples of nucleosomes. We also checked the TSS enrichment score to evaluate the ATAC‐seq data. The TSS enrichment calculation is a signal‐to‐noise calculation. If there is a high read signal at the TSS (highly open region of the genome), there should be an increase in signal up to a peak in the middle. We used the signal value at the center of the distribution after this normalization as our TSS enrichment metric.

Peaks on replicates and self‐pseudoreplicates were called using MACS2 (Zhang et al., 2008) (‐‐nomodel ‐‐extsize 200 ‐ shift ‐ 100). Peaks in each sample were called for QC purposes. To identify the most high‐confidence set of peaks, we employed a strategy using peaks overlapping across replicates and peaks overlapping between samples and pseudoreplicates from each sample. These were generally high to medium confidence peaks. We ran an IDR assessment on peaks between each sample and self‐pseudoreplicates with an IDR threshold of 0.05.

All called peaks from all samples were merged to acquire a consensus peak set. For each consensus peak, its enrichment was defined as the ATAC signal intensity (normalized read count per base) subtracted by the background noise (normalized read count per base). R package Diffbind was utilized to detect potential differentially accecible regions (DARs) from the consensus peak set. Statistically significant DARs were defined as peaks with Log_2_ fold enrichment > 0.5 and false discovery rate < 0.05. Motif analysis on DARs was performed using the MEME suite (Heinz et al., 2010) with default settings. Motifs were only kept when the *P*‐value was < 0.01. Gene Ontology enrichment was performed using R package “ClusterProfiler” with default settings. All sequencing tracks were viewed using the IGV (2.3.61).

### High‐throughput/resolution chromosome conformation capture library generation and data analysis

High‐throughput/resolution 3C libraries were constructed according to a previous study (Crémazy et al., 2018). Briefly, samples were cross‐linked under vacuum infiltration for 30 min with 3% formaldehyde at 4°C and quenched with 0.375 mol/L glycine for 5 min. The cross‐linked samples were subsequently lysed. Endogenous nucleases were inactivated with 0.3% sodium dodecyl sulfate, and chromatin DNA was digested using 100 U MboI (NEB), marked with biotin‐14‐dCTP (Invitrogen, Carlsbad, CA, USA), and then ligated with 50 U T4 DNA ligase (NEB). After reversing cross‐links, the ligated DNA was extracted using a QIAamp DNA Mini Kit (Qiagen, Redwood City, CA, USA) according to the manufacturers' instructions. Purified DNA was sheared to 300–500 bp fragments and further blunt‐end repaired, A‐tailed, and adaptor‐added; it was then purified through biotin‐streptavidin–mediated pull‐down and PCR amplification. Finally, the Hi‐C libraries were quantified and sequenced on an Illumina Nova‐seq platform (San Diego, CA, USA) or MGI‐seq platform (BGI, Shenzhen City, China).

### Interaction matrix construction

Trimmomatics (version 0.39) was used to obtain clean reads, with the parameters ILLUMINACLIP:adapter.fa:2:30:10:8:true LEADING:3TRAILING:3 SLIDINGWINDOW:4:15 MINLEN:36. Following the 4DN Hi‐C data analysis pipeline, the clean reads were aligned to the reference genome using bwa‐SP5M (bwa version 0.7.17), and then the alignment results were converted to the pair format proposed by 4DN using pairtools parse, sort, and dedup (pairtools version 0.3.0), with default parameters. If there were multiple samples with biological replicates, GenomeDISCO (Ursu et al., 2018) was used to evaluate the correlation between the biological replicates of each sample (integrated into 3DChromatin_ReplicateQC, http://github.com/kundajelab/3DChromatin_ReplicateQC), and the reproducibility analysis heatmap was plotted. The alignment results of the biological replicates with high reproducibility were merged to maximize sample resolution. The highest achievable resolution was evaluated after merging the samples by following the method proposed by the first *in situ* Hi‐C paper (Rao et al., 2014). Based on the evaluation results, cooler cload (cooler version 0.8.11) was used to construct the matrix from the alignment results, with resolutions including 200, 100, 40, and 10 kb, and then cooler balance was used to balance the matrix. At the same time, a cooler was used to calculate and plot the interaction frequency decay curve with the interaction distance, that is, P (s) curve.

### Compartment identification

Compartment detection was performed using PCA dimensionality reduction, which originated from the first Hi‐C paper (Lieberman‐Aiden et al., 2009). Specifically, the balanced 100‐kb resolution matrix was used and calculated as the input for cooltools eigs‐cis. After obtaining the first eigenvector using PCA dimensionality reduction, the results were corrected with gene density information. Regions with high gene density corresponded to Compartment A, and regions with low gene density corresponded to Compartment B (Ke et al., 2017).

### Topologically associated domain identification

The insulation score method was used to detect TADs, which was originally proposed by Crane et al. (2015), and later optimized with Open2C integrating this method into cooltools. To detect TAD structures from the balanced 40‐kb resolution matrix, cooltools insulation (cooltools version 0.5.1) was used with the following parameters: ‐‐ignore‐diags 2 ‐‐threshold 0.1 ‐‐min‐dist‐bad‐bin 5 (Cha et al., 2021; Wang et al., 2022).

### Loop identification

To minimize the impact of resolution on loop detection, mustache (version v1.0.1) was used to detect loops from 5‐, 10‐, and 20‐kb resolution matrices following the method in Hsieh's study (Hsieh et al., 2022). The loops from the above resolutions were then merged, taking the highest resolution result when the loops overlapped.

### Differential interaction matrix

Before comparing the interaction matrices of different samples, the matrices were normalized and then compared. The LOWESS *z*‐score method (Crane et al., 2015) was used to process the 100‐kb resolution matrix, and then the normalized matrix of the treatment group was subtracted from the control group to obtain the difference matrix, which was then displayed in the form of a heatmap. In the figure, positive values indicate sites where the interaction of the treatment group was enhanced relative to the control group, and negative values indicate sites where the interaction of the treatment group was weakened relative to the control group.

### Compartment comparison

When the compartment of some bins changed, it implied a change in chromatin activity. After obtaining the compartment classification results of each bin for the two samples, the corresponding bins of the two samples were compared, and four change patterns were obtained: A2A, A2B, B2A, and B2B. For example, A2B means that the bin is Compartment A in the control group sample and compartment B in the treatment group; that is, after treatment, the bin changes from the active to the repressive state. Saddle plot analysis was performed on the two samples, as proposed by Schwarzer et al. (2017). Cooltools saddle combined the results of the first eigenvector PC1 to integrate and quantify the interactions between A‐A and B‐B, with the following parameters: cooltools saddle‐‐contact‐type cis‐‐qrange 0.02 0.98. To quantify the interactions between A‐A and B‐B, the top 20% of regions with the highest A‐A interaction strength were selected, that is, top (A‐A), and the top 20% of regions with the highest B‐B interaction strength, that is, top (B‐B). The bottom 20% of regions with the lowest A‐B interaction strength, that is, bottom (A‐B), were used to normalize top A‐A and top B‐B. The following formula was used for this calculation: *y* = top (B‐B)/bottom (A‐B); *x* = top (A‐A)/bottom (A‐B). The calculation of compartment saddle strength is based on Akgol's research (Akgol et al., 2021).

### Topologically associated domain comparison

Topologically associated domain comparison was divided into two parts: one focused on the TAD boundary, and the other focused on the TAD body. Based on the TAD boundary comparison, the TAD boundaries of the two samples overlapped, and the unique boundaries of each sample and the common boundaries were obtained and plotted in a Venn diagram. Then, coolpup.py was used to perform a TAD boundary aggregate analysis on the unique and common boundaries of the two samples. To obtain the gene enrichment results on the sample‐specific boundaries, clusterProfiler was used to perform GO/KEGG enrichment analysis on these overlapping genes. Based on TAD body comparison, the TAD bodies of the two samples overlapped following the method in Yuwen's study (Ke et al., 2017). If two or more TADs in the control group corresponded to one TAD in the treatment group, it was called a merge; if one TAD in the control group corresponded to two or more TADs in the treatment group, it was called a split. If one TAD in the control group overlapped with one TAD in the treatment group by more than 75% in both samples, it was called stable; the rest were called rearrangements. Subsequently, coolpup.py was used to perform TAD body aggregate analysis on each region.

### Loop comparison

Considering that the loop sites of the two samples may have some offsets, the loops of the two samples were overlapped following the method of Hongbo's study (Yang et al., 2020), with parameter bedtools pairtopair‐type both‐f 0.5 to detect the common loops of the two samples and bedtools pairtopair‐type notboth‐slop 40,000 to detect the sample‐specific loops. Based on this, coolpup.py was used to perform aggregate peak analysis according to Rao et al. (2014).

### Whole genome bisulfite sequencing and analysis

Whole genome bisulfite sequencing was performed by Frasergen (Wuhan, China). Genomic DNA was interrupted to an appropriate range (300–500 bp), and the ends of repaired DNA fragments were flattened. After bisulfite treatment, the DNA was purified and recovered for PCR amplification. The amplified library was purified for quality inspection, and the qualified library was sequenced (Krueger and Andrews, 2011). Bismark software was used to compare the filtered data with the reference sequence, and the dmrseq software package was used for DMRs analysis. The obtained DMRs candidate regions were filtered (Yu et al., 2012) using a filtering standard of *Q* value ≤ 0.05.

### Plasmid construction and peanut hairy root transformation

The *AhMsrA* coding sequence (CDS) was amplified and inserted into 5'‐Hind III and 3'‐SalI cloning sites of pCAMBIA1300‐GFP using Golden Gate Assembly to construct the *AhMsrA*‐OE overexpression plasmid. The target sequences of *AhMsrA* CDS were constructed into the pK7GWIWG2D(II) vector using a LR reaction to generate the *AhMsrA*‐KD silencing construct. For CRISPR knockdown vector construction, gRNAs were designed using Crispr‐P (http://crispr.hzau.edu.cn/CRISPR2/) software. Two single‐guide RNAs (sgRNAs) with high scores were selected and cloned into the pKSE401‐GFP vector using vector pCBC‐DT1T2 as a template to generate the *AhMsrA*‐KO construct.

*Agrobacterium rhizogenes* strain K599 was applied to conduct peanut hairy root transformation. K599 solution carrying the test plasmids was used to inoculate peanut seedlings at 3 d after germination at a cut site in the hypocotyl. Peanut seedlings were transferred into pots filled with vermiculite and grown in a growth chamber.

## CONFLICTS OF INTEREST

The authors declare no conflicts of interest.

## AUTHOR CONTRIBUTIONS

L.X.W., and Z.S.K. designed the experiments; L.X.W., S.Q.H., X.R.Z., and C.H.M. performed the experiments; L.X.W., C.H.M., B.J.N., G.Y.J., T.Y., X.L., and M.R. analyzed the data; L.X.W. wrote the manuscript; C.H.M., and Z.S.K. revised the manuscript. All authors read and approved the manuscript.

## Supporting information

Additional Supporting Information may be found online in the supporting information tab for this article: http://onlinelibrary.wiley.com/doi/10.1111/jipb.70007/suppinfo

**Figure S1.** Three‐dimensional model of whole chromosomes in peanut roots and nodules
**Figure S2.** Single‐chromosome interactions in peanut roots and nodules at a 400‐kb resolution
**Figure S3.** Length distribution of genomic compartments
**Figure S4.** Distribution of gene count and GC content in GCs
**Figure S5.** Functional annotation of genes associated with Compartment A/B transitions
**Figure S6.** Correlation analysis of ATAC‐seq data
**Figure S7.** Characterization of OCRs and epigenome markers in peanut roots
**Figure S8.** Screening of differentially expressed genes
**Figure S9.** GO enrichment results for DEGs from the Nodule versus Root comparison
**Figure S10.** KEGG enrichment results for DEGs from the Nodule versus Root comparison
**Figure S11.** Number of root‐ and nodule‐enriched genes associated with different chromatin features that are differentially expressed between roots and nodules
**Figure S12.** Nodule‐enriched differentially expressed genes and chromatin states in roots and nodules
**Figure S13.** KEGG enrichment analysis of genes located in the TAD boundary of the nodules
**Figure S14.** KEGG enrichment analysis of genes located in the insulation region of the nodules
**Figure S15.** Association study of TADs border region and with epigenetic modifications
**Figure S16.** Example of differentially presented loops around the *ahNIN* gene that was highly expressed in root nodules
**Figure S17.** Analysis of the chromatin loop around the *ahNIN*
**Figure S18.** Diagram of the genomic information of the differentially presented loop around the *AhMsrA* gene that is highly expressed in root nodules
**Figure S19.** Phenotypic analysis of *AhMsrA*‐RNA interference in peanut nodulation
**Figure S20.** Gene editing status of *AhMsrA* knockout roots
**Figure S21.** Schematic diagram of enhancer identification in peanut roots and root nodules
**Figure S22.** Enrichment analysis of interaction genes with gain enhancers in nodule Hi‐C compared with root Hi‐C
**Figure S23.** OCRs and nodule‐enriched genes associated with nodule‐specific enhancers

**Table S1.** Hi‐C sequencing data generated for peanut root and root nodules
**Table S2.** Summary of the sequencing data of ATAC‐seq
**Table S3.** Summary of the sequencing data of ChIP‐seq
**Table S4.** Summary of the sequencing data of WGBS
**Table S5.** Statistics of RNA sequencing data quality
**Table S6.** Distribution of compartment A and B on each chromosome in root and nodule genomes
**Table S7.** Genome‐wide distribution of TAD on 20 chromosomes of peanut genome
**Table S8.** Open chromatin regions in nodule
**Table S9.** Differential OCRs
**Table S10.** List of genes associated with NRE contained LoOCRs
**Table S11.** Differentially expressed genes with their changed epigenetic features in root and nodule
**Table S12.** Genome‐wide distribution of *cis*‐interactions on chromosomes
**Table S13.** The top 10 motifs enriched around the TAD boundaries
**Table S14.** List of genes located distributed in the TAD boundary
**Table S15.** Chromatin accessibility and epigenetic modifications in TAD
**Table S16.** Known nodulation‐related genes located in the loop region
**Table S17.** List of primers for used 3C‐qPCR study
**Table S18.** Differential H3K27me3
**Table S19.** dOCR‐gene loops in nodules
**Table S20.** Nodule‐active dOCR‐gene loops
**Table S21.** Chromatin loops in nodules
**Table S22.** Genes distribution of peanut altered chromatin loops
**Table S23.** H3K4me3 associated enhancers
**Table S24.** H3K9ac associated enhancers
**Table S25.** Nodule‐specific enhancers

## Data Availability

The sequencing data sets of peanut roots and root nodules for this project have been submitted to the National Genomics Data Center, under accession number CRA019413. Sequence information about *AhNIN*, *AhMsrA*, *AhNSP2*, *AhNFYA1*, *AhNFRe*, *AhNOOT1*, *AhSYMREM1*, *AhCNGC15b*, *AhSUS1*, *AhNRT1.1*, *Ahnodulin MtN21*, *AhERN1*, *AhMATE1*, *Ah*Sulfite exporter, *Ahsyntaxin* and *Ahankyrin* mentioned in this paper can be found in the peanut database (https://www.peanutbase.org/tools/search/gene.html). The accession numbers are as follows: AhNIN (I65W25), *AhMsrA* (4A36F8), *AhNSP2* (JU87H7), *AhSUS1* (4VZ14Z), *AhNFYA1* (EWBI0W), *AhNFRe* (W1BJPM), *AhNOOT1*(A4KSPL), *AhSYMREM1* (XJXR2L), *AhNRT1.1* (LZ85EL), *Ahnodulin MtN21* (L4R73E), *AhERN1* (J0DG7J), *AhMATE1* (HSA1AU), *Ah*Sulfite exporter (FYY1I0), *Ahankyrin* (4YMK55), *Ahsyntaxin* (IINC9K) and *AhCNGC15b* (19E8GC).

## References

[jipb70007-bib-0001] Akgol, O.B. , Yang, L. , Abraham, S. , Venev, S.V. , Krietenstein, N. , Parsi, K.M. , Ozadam, H. , Oomen, M.E. , Nand, A. , Mao, H. , et al. (2021). Systematic evaluation of chromosome conformation capture assays. Nat. Methods 18: 1046–1055. 34480151 10.1038/s41592-021-01248-7 PMC8446342

[jipb70007-bib-0002] Andersson, R. , and Sandelin, A. (2020). Determinants of enhancer and promoter activities of regulatory elements. Nat. Rev. Genet. 21: 71–87. 31605096 10.1038/s41576-019-0173-8

[jipb70007-bib-0003] Andrews, S. (2014). FastQC a quality control tool for high throughput sequence data. Available online at: http://www.bioinformatics.babraham.ac.uk/projects/fastqc

[jipb70007-bib-0004] Barutcu, A.R. , Lajoie, B.R. , McCord, R.P. , Tye, C.E. , Hong, D. , Messier, T.L. , Browne, G. , van Wijnen, A.J. , Lian, J.B. , Stein, J.L. , et al. (2015). Chromatin interaction analysis reveals changes in small chromosome and telomere clustering between epithelial and breast cancer cells. Genome Biol. 16: 214. 26415882 10.1186/s13059-015-0768-0 PMC4587679

[jipb70007-bib-0005] Beagan, J.A. , and Phillips‐Cremins, J.E. (2020). On the existence and functionality of topologically associating domains. Nat. Genet. 52: 8–16. 31925403 10.1038/s41588-019-0561-1 PMC7567612

[jipb70007-bib-0006] Berke, L. , Sanchez‐Perez, G.F. , and Snel, B. (2012). Contribution of the epigenetic mark H3K27me3 to functional divergence after whole genome duplication in Arabidopsis. Genome Biol. 13: R94. 23034476 10.1186/gb-2012-13-10-r94 PMC3491422

[jipb70007-bib-0008] Bhattacharjee, O. , Raul, B. , Ghosh, A. , Bhardwaj, A. , Bandyopadhyay, K. , and Sinharoy, S. (2022). Nodule INception‐independent epidermal events lead to bacterial entry during nodule development in peanut (*Arachis hypogaea*). New Phytol. 236: 2265–2281. 36098671 10.1111/nph.18483

[jipb70007-bib-0009] Bolger, A.M. , Lohse, M. , and Usadel, B. (2014). Trimmomatic: A flexible trimmer for Illumina sequence data. Bioinformatics (Oxford, England) 30: 2114–2120. 24695404 10.1093/bioinformatics/btu170 PMC4103590

[jipb70007-bib-0010] Bozsoki, Z. , Gysel, K. , Hansen, S.B. , Lironi, D. , Krönauer, C. , Feng, F. , de Jong, N. , Vinther, M. , Kamble, M. , Thygesen, M.B. , et al. (2020). Ligand‐recognizing motifs in plant LysM receptors are major determinants of specificity. Science (New York, N.Y.) 369: 663–670. 32764065 10.1126/science.abb3377

[jipb70007-bib-0011] Canfield, D.E. , Glazer, A.N. , and Falkowski, P.G. (2010). The evolution and future of Earth's nitrogen cycle. Science (New York, N.Y.) 330: 192–196. 20929768 10.1126/science.1186120

[jipb70007-bib-0012] Cha, H.J. , Uyan, Ö. , Y., Liu, T. , Zhu, Q. , Tothova, Z. , Botten, G.A. , Xu, J. , Yuan, G.C. , Dekker, J. , et al. (2021). Inner nuclear protein Matrin‐3 coordinates cell differentiation by stabilizing chromatin architecture. Nat. Commun. 12: 6241. 34716321 10.1038/s41467-021-26574-4 PMC8556400

[jipb70007-bib-0013] Chakraborty, C. , Nissen, I. , Vincent, C.A. , Hägglund, A.C. , Hörnblad, A. , and Remeseiro, S. (2023). Rewiring of the promoter‐enhancer interactome and regulatory landscape in glioblastoma orchestrates gene expression underlying neurogliomal synaptic communication. Nat. Commun. 14: 6446. 37833281 10.1038/s41467-023-41919-x PMC10576091

[jipb70007-bib-0014] Charpentier, M. , Sun, J. , Vaz Martins, T. , Radhakrishnan, G.V. , Findlay, K. , Soumpourou, E. , Thouin, J. , Véry, A.A. , Sanders, D. , Morris, R.J. , et al. (2016). Nuclear‐localized cyclic nucleotide‐gated channels mediate symbiotic calcium oscillations. Science (New York, N.Y.) 352: 1102–1105. 27230377 10.1126/science.aae0109

[jipb70007-bib-0015] Crane, E. , Bian, Q. , McCord, R.P. , Lajoie, B.R. , Wheeler, B.S. , Ralston, E.J. , Uzawa, S. , Dekker, J. , and Meyer, B.J. (2015). Condensin‐driven remodelling of X chromosome topology during dosage compensation. Nature 523: 240–244. 26030525 10.1038/nature14450 PMC4498965

[jipb70007-bib-0016] Crémazy, F.G. , Rashid, F.M. , Haycocks, J.R. , Lamberte, L.E. , Grainger, D.C. , and Dame, R.T. (2018). Determination of the 3D genome organization of bacteria using Hi‐C. Methods Mol. Biol (Clifton, N.J.). 1837: 3–18. 10.1007/978-1-4939-8675-0_1 30109602

[jipb70007-bib-0017] Dong, P. , Tu, X. , Chu, P.Y. , Lü, P. , Zhu, N. , Grierson, D. , Du, B. , Li, P. , and Zhong, S. (2017). 3D chromatin architecture of large plant genomes determined by local A/B compartments. Mol. Plant 10: 1497–1509. 29175436 10.1016/j.molp.2017.11.005

[jipb70007-bib-0018] Feng, J. , Liu, T. , Qin, B. , Zhang, Y. , and Liu, X.S. (2012). Identifying ChIP‐seq enrichment using MACS. Nat. Protoc. 7: 1728–1740. 22936215 10.1038/nprot.2012.101 PMC3868217

[jipb70007-bib-0019] Ferguson, B.J. , Mens, C. , Hastwell, A.H. , Zhang, M. , Su, H. , Jones, C.H. , Chu, X. , and Gresshoff, P.M. (2019). Legume nodulation: The host controls the party. Plant Cell Environ. 42: 41–51. 29808564 10.1111/pce.13348

[jipb70007-bib-0020] Fortin, J.P. , and Hansen, K.D. (2015). Reconstructing A/B compartments as revealed by Hi‐C using long‐range correlations in epigenetic data. Genome Biol. 16: 180. 26316348 10.1186/s13059-015-0741-y PMC4574526

[jipb70007-bib-0021] Garg, V. , Dudchenko, O. , Wang, J. , Khan, A.W. , Gupta, S. , Kaur, P. , Han, K. , Saxena, R.K. , Kale, S.M. , Pham, M. , et al. (2022). Chromosome‐length genome assemblies of six legume species provide insights into genome organization, evolution, and agronomic traits for crop improvement. J. Adv. Res. 42: 315–329. 36513421 10.1016/j.jare.2021.10.009 PMC9788938

[jipb70007-bib-0022] Ghantasala, S. , and Roy Choudhury, S. (2022). Nod factor perception: An integrative view of molecular communication during legume symbiosis. Plant Mol. Biol. 110: 485–509. 36040570 10.1007/s11103-022-01307-3

[jipb70007-bib-0023] Ghavi‐Helm, Y. , Jankowski, A. , Meiers, S. , Viales, R.R. , Korbel, J.O. , and Furlong, E.E.M. (2019). Highly rearranged chromosomes reveal uncoupling between genome topology and gene expression. Nat. Genet. 51: 1272–1282. 31308546 10.1038/s41588-019-0462-3 PMC7116017

[jipb70007-bib-0024] Griesmann, M. , Chang, Y. , Liu, X. , Song, Y. , Haberer, G. , Crook, M.B. , Billault‐Penneteau, B. , Lauressergues, D. , Keller, J. , Imanishi, L. , et al. (2018). Phylogenomics reveals multiple losses of nitrogen‐fixing root nodule symbiosis. Science 361 (6398): eaat1743. 29794220 10.1126/science.aat1743

[jipb70007-bib-0025] Guo, L. , Cao, X. , Liu, Y. , Li, J. , Li, Y. , Li, D. , Zhang, K. , Gao, C. , Dong, A. , and Liu, X. (2018). A chromatin loop represses WUSCHEL expression in Arabidopsis. Plant J. 94: 1083–1097. 29660180 10.1111/tpj.13921

[jipb70007-bib-0026] Heinz, S. , Benner, C. , Spann, N. , Bertolino, E. , Lin, Y.C. , Laslo, P. , Cheng, J.X. , Murre, C. , Singh, H. , and Glass, C.K. (2010). Simple combinations of lineage‐determining transcription factors prime cis‐regulatory elements required for macrophage and B cell identities. Mol. Cell 38: 576–589. 20513432 10.1016/j.molcel.2010.05.004 PMC2898526

[jipb70007-bib-0027] Hoang, N.T. , Tóth, K. , and Stacey, G. (2020). The role of microRNAs in the legume‐rhizobium nitrogen‐fixing symbiosis. J. Exp. Bot. 71: 1668–1680. 32163588 10.1093/jxb/eraa018

[jipb70007-bib-0028] Hossain, M.S. , Shrestha, A. , Zhong, S. , Miri, M. , Austin, R.S. , Sato, S. , Ross, L. , Huebert, T. , Tromas, A. , Torres‐Jerez, I. , et al. (2016). *Lotus japonicus* NF‐YA1 plays an essential role during nodule differentiation and targets members of the SHI/STY gene family. Mol. Plant Microbe Interact. 29: 950–964. 27929718 10.1094/MPMI-10-16-0206-R

[jipb70007-bib-0029] Hsieh, T.S. , Cattoglio, C. , Slobodyanyuk, E. , Hansen, A.S. , Darzacq, X. , and Tjian, R. (2022). Enhancer‐promoter interactions and transcription are largely maintained upon acute loss of CTCF, cohesin, WAPL or YY1. Nat. Genet. 54: 1919–1932. 36471071 10.1038/s41588-022-01223-8 PMC9729117

[jipb70007-bib-0030] Kaló, P. , Gleason, C. , Edwards, A. , Marsh, J. , Mitra, R.M. , Hirsch, S. , Jakab, J. , Sims, S. , Long, S.R. , Rogers, J. , et al. (2005). Nodulation signaling in legumes requires NSP2, a member of the GRAS family of transcriptional regulators. Science (New York, N.Y.) 308: 1786–1789. 15961668 10.1126/science.1110951

[jipb70007-bib-0031] Ke, Y. , Xu, Y. , Chen, X. , Feng, S. , Liu, Z. , Sun, Y. , Yao, X. , Li, F. , Zhu, W. , Gao, L. , et al. (2017). 3D Chromatin structures of mature gametes and structural reprogramming during mammalian embryogenesis. Cell 170: 367–381.e320. 28709003 10.1016/j.cell.2017.06.029

[jipb70007-bib-0032] Krueger, F. , and Andrews, S.R. (2011). Bismark: A flexible aligner and methylation caller for bisulfite‐Seq applications. Bioinformatics (Oxford, England) 27: 1571–1572. 21493656 10.1093/bioinformatics/btr167 PMC3102221

[jipb70007-bib-0033] Langmead, B. , and Salzberg, S.L. (2012). Fast gapped‐read alignment with Bowtie 2. Nat. Methods 9: 357–359. 22388286 10.1038/nmeth.1923 PMC3322381

[jipb70007-bib-0034] Lefebvre, B. , Timmers, T. , Mbengue, M. , Moreau, S. , Hervé, C. , Tóth, K. , Bittencourt‐Silvestre, J. , Klaus, D. , Deslandes, L. , Godiard, L. , et al. (2010). A remorin protein interacts with symbiotic receptors and regulates bacterial infection. Proc. Natl. Acad. Sci. U.S.A. 107: 2343–2348. 20133878 10.1073/pnas.0913320107 PMC2836688

[jipb70007-bib-0035] Li, H. , Handsaker, B. , Wysoker, A. , Fennell, T. , Ruan, J. , Homer, N. , Marth, G. , Abecasis, G. , and Durbin, R. (2009). The sequence alignment/Map format and SAMtools. Bioinformatics (Oxford, England) 25: 2078–2079. 19505943 10.1093/bioinformatics/btp352 PMC2723002

[jipb70007-bib-0036] Li, P. , and Leonard, W.J. (2018). Chromatin accessibility and interactions in the transcriptional regulation of T cells. Front. Immunol. 9: 2738. 30524449 10.3389/fimmu.2018.02738 PMC6262064

[jipb70007-bib-0037] Li, Z. , Sun, L. , Xu, X. , Liu, Y. , He, H. , and Deng, X.W. (2024). Light control of three‐dimensional chromatin organization in soybean. Plant Biotechnol. J. 22: 2596–2611. 38762905 10.1111/pbi.14372 PMC11331798

[jipb70007-bib-0038] Lieberman‐Aiden, E. , van Berkum, N.L. , Williams, L. , Imakaev, M. , Ragoczy, T. , Telling, A. , Amit, I. , Lajoie, B.R. , Sabo, P.J. , Dorschner, M.O. , et al. (2009). Comprehensive mapping of long‐range interactions reveals folding principles of the human genome. Science (New York, N.Y.) 326: 289–293. 19815776 10.1126/science.1181369 PMC2858594

[jipb70007-bib-0039] Liu, C. , Cheng, Y.J. , Wang, J.W. , and Weigel, D. (2017). Prominent topologically associated domains differentiate global chromatin packing in rice from Arabidopsis. Nat. Plants 3: 742–748. 28848243 10.1038/s41477-017-0005-9

[jipb70007-bib-0040] Liu, J. , Rutten, L. , Limpens, E. , van der Molen, T. , van Velzen, R. , Chen, R. , Chen, Y. , Geurts, R. , Kohlen, W. , Kulikova, O. , et al. (2019). A remote cis‐regulatory region is required for NIN expression in the pericycle to initiate nodule primordium formation in *Medicago truncatula* . Plant Cell 31: 68–83. 30610167 10.1105/tpc.18.00478 PMC6391699

[jipb70007-bib-0041] Liu, Z. , Kong, X. , Long, Y. , Liu, S. , Zhang, H. , Jia, J. , Cui, W. , Zhang, Z. , Song, X. , Qiu, L. , et al. (2023). Integrated single‐nucleus and spatial transcriptomics captures transitional states in soybean nodule maturation. Nat. Plants 9: 515–524. 37055554 10.1038/s41477-023-01387-z

[jipb70007-bib-0042] Luo, Z. , Liu, H. , and Xie, F. (2023). Cellular and molecular basis of symbiotic nodule development. Curr. Opin. Plant Biol. 76: 102478. 37857037 10.1016/j.pbi.2023.102478

[jipb70007-bib-0043] Magne, K. , Couzigou, J.M. , Schiessl, K. , Liu, S. , George, J. , Zhukov, V. , Sahl, L. , Boyer, F. , Iantcheva, A. , Mysore, K.S. , et al. (2018). MtNODULE ROOT1 and MtNODULE ROOT2 are essential for indeterminate nodule identity. Plant Physiol. 178: 295–316. 30026291 10.1104/pp.18.00610 PMC6130032

[jipb70007-bib-0044] Murakami, E. , Cheng, J. , Gysel, K. , Bozsoki, Z. , Kawaharada, Y. , Hjuler, C.T. , Sørensen, K.K. , Tao, K. , Kelly, S. , Venice, F. , et al. (2018). Epidermal LysM receptor ensures robust symbiotic signalling in *Lotus japonicus* . eLife 7: e33506. 29957177 10.7554/eLife.33506 PMC6025957

[jipb70007-bib-0045] Nagymihály, M. , Veluchamy, A. , Györgypál, Z. , Ariel, F. , Jégu, T. , Benhamed, M. , Szűcs, A. , Kereszt, A. , Mergaert, P. , and Kondorosi, É. (2017). Ploidy‐dependent changes in the epigenome of symbiotic cells correlate with specific patterns of gene expression. Proc. Natl. Acad. Sci. U.S.A. 114: 4543–4548. 28404731 10.1073/pnas.1704211114 PMC5410778

[jipb70007-bib-0046] Nguyen, C.X. , Dohnalkova, A. , Hancock, C.N. , Kirk, K.R. , Stacey, G. , and Stacey, M.G. (2023). Critical role for uricase and xanthine dehydrogenase in soybean nitrogen fixation and nodule development. Plant Genome 16: e20171. 34904377 10.1002/tpg2.20172 PMC12807372

[jipb70007-bib-0047] Ni, L. , Liu, Y. , Ma, X. , Liu, T. , Yang, X. , Wang, Z. , Liang, Q. , Liu, S. , Zhang, M. , Wang, Z. , et al. (2023). Pan‐3D genome analysis reveals structural and functional differentiation of soybean genomes. Genome Biol. 24: 12. 36658660 10.1186/s13059-023-02854-8 PMC9850592

[jipb70007-bib-0048] Oka, R. , Zicola, J. , Weber, B. , Anderson, S.N. , Hodgman, C. , Gent, J.I. , Wesselink, J.J. , Springer, N.M. , Hoefsloot, H.C.J. , Turck, F. , et al. (2017). Genome‐wide mapping of transcriptional enhancer candidates using DNA and chromatin features in maize. Genome Biol. 18: 137. 28732548 10.1186/s13059-017-1273-4 PMC5522596

[jipb70007-bib-0049] Oldroyd, G.E. , and Downie, J.A. (2008). Coordinating nodule morphogenesis with rhizobial infection in legumes. Annu. Rev. Plant Biol. 59: 519–546. 18444906 10.1146/annurev.arplant.59.032607.092839

[jipb70007-bib-0050] Peng, Y. , Xiong, D. , Zhao, L. , Ouyang, W. , Wang, S. , Sun, J. , Zhang, Q. , Guan, P. , Xie, L. , Li, W. , et al. (2019). Chromatin interaction maps reveal genetic regulation for quantitative traits in maize. Nat. Commun. 10: 2632. 31201335 10.1038/s41467-019-10602-5 PMC6572838

[jipb70007-bib-0051] Peng, Z. , Chen, H. , Tan, L. , Shu, H. , Varshney, R.K. , Zhou, Z. , Zhao, Z. , Luo, Z. , Chitikineni, A. , Wang, L. , et al. (2021). Natural polymorphisms in a pair of NSP2 homoeologs can cause loss of nodulation in peanut. J. Exp. Bot. 72: 1104–1118. 33130897 10.1093/jxb/eraa505

[jipb70007-bib-0052] Peng, Z. , Liu, F. , Wang, L. , Zhou, H. , Paudel, D. , Tan, L. , Maku, J. , Gallo, M. , and Wang, J. (2017). Transcriptome profiles reveal gene regulation of peanut (*Arachis hypogaea* L.) nodulation. Sci. Rep. 7: 40066. 28059169 10.1038/srep40066 PMC5216375

[jipb70007-bib-0053] Quilbé, J. , Montiel, J. , Arrighi, J.F. , and Stougaard, J. (2022). Molecular mechanisms of intercellular rhizobial infection: Novel findings of an ancient process. Front. Plant Sci. 13: 922982. 35812902 10.3389/fpls.2022.922982 PMC9260380

[jipb70007-bib-0054] Ramírez, F. , Ryan, D.P. , Grüning, B. , Bhardwaj, V. , Kilpert, F. , Richter, A.S. , Heyne, S. , Dündar, F. , and Manke, T. (2016). deepTools2: A next generation web server for deep‐sequencing data analysis. Nucleic Acids Res. 44: W160–W165. 27079975 10.1093/nar/gkw257 PMC4987876

[jipb70007-bib-0055] Rao, S.S. , Huntley, M.H. , Durand, N.C. , Stamenova, E.K. , Bochkov, I.D. , Robinson, J.T. , Sanborn, A.L. , Machol, I. , Omer, A.D. , Lander, E.S. , et al. (2014). A 3D map of the human genome at kilobase resolution reveals principles of chromatin looping. Cell 159: 1665–1680. 25497547 10.1016/j.cell.2014.11.021 PMC5635824

[jipb70007-bib-0056] Rodgers‐Melnick, E. , Vera, D.L. , Bass, H.W. , and Buckler, E.S. (2016). Open chromatin reveals the functional maize genome. Proc. Natl. Acad. Sci. U.S.A. 113: E3177–E3184. 27185945 10.1073/pnas.1525244113 PMC4896728

[jipb70007-bib-0057] Roy, S. , Liu, W. , Nandety, R.S. , Crook, A. , Mysore, K.S. , Pislariu, C.I. , Frugoli, J. , Dickstein, R. , and Udvardi, M.K. (2020). Celebrating 20 years of genetic discoveries in legume nodulation and symbiotic nitrogen fixation. Plant Cell 32: 15–41. 31649123 10.1105/tpc.19.00279 PMC6961631

[jipb70007-bib-0058] Satgé, C. , Moreau, S. , Sallet, E. , Lefort, G. , Auriac, M.C. , Remblière, C. , Cottret, L. , Gallardo, K. , Noirot, C. , Jardinaud, M.F. , et al. (2016). Reprogramming of DNA methylation is critical for nodule development in *Medicago truncatula* . Nat. Plants 2: 16166. 27797357 10.1038/nplants.2016.166

[jipb70007-bib-0059] Schauser, L. , Roussis, A. , Stiller, J. , and Stougaard, J. (1999). A plant regulator controlling development of symbiotic root nodules. Nature 402: 191–195. 10647012 10.1038/46058

[jipb70007-bib-0060] Schwarzer, W. , Abdennur, N. , Goloborodko, A. , Pekowska, A. , Fudenberg, G. , Loe‐Mie, Y. , Fonseca, N.A. , Huber, W. , Haering, C.H. , Mirny, L. , et al. (2017). Two independent modes of chromatin organization revealed by cohesin removal. Nature 551: 51–56. 29094699 10.1038/nature24281 PMC5687303

[jipb70007-bib-0061] Shu, H. , Luo, Z. , Peng, Z. , and Wang, J. (2020). The application of CRISPR/Cas9 in hairy roots to explore the functions of AhNFR1 and AhNFR5 genes during peanut nodulation. BMC Plant Biol. 20: 417. 32894045 10.1186/s12870-020-02614-x PMC7487912

[jipb70007-bib-0062] Sinharoy, S. , and DasGupta, M. (2009). RNA interference highlights the role of CCaMK in dissemination of endosymbionts in the Aeschynomeneae legume Arachis. Mol. Plant Microbe Interact. 22: 1466–1475. 19810815 10.1094/MPMI-22-11-1466

[jipb70007-bib-0063] Sotelo‐Silveira, M. , Chávez Montes, R.A. , Sotelo‐Silveira, J.R. , Marsch‐Martínez, N. , and de Folter, S. (2018). Entering the next dimension: Plant genomes in 3D. Trends Plant Sci. 23: 598–612. 29703667 10.1016/j.tplants.2018.03.014

[jipb70007-bib-0064] Sun, Y. , Dong, L. , Zhang, Y. , Lin, D. , Xu, W. , Ke, C. , Han, L. , Deng, L. , Li, G. , Jackson, D. , et al. (2020). 3D genome architecture coordinates trans and cis regulation of differentially expressed ear and tassel genes in maize. Genome Biol. 21: 143. 32546248 10.1186/s13059-020-02063-7 PMC7296987

[jipb70007-bib-0065] Ursu, O. , Boley, N. , Taranova, M. , Wang, Y.X.R. , Yardimci, G.G. , Stafford Noble, W. , and Kundaje, A. (2018). GenomeDISCO: A concordance score for chromosome conformation capture experiments using random walks on contact map graphs. Bioinformatics (Oxford, England) 34: 2701–2707. 29554289 10.1093/bioinformatics/bty164 PMC6084597

[jipb70007-bib-0066] Wang, C. , Liu, C. , Roqueiro, D. , Grimm, D. , Schwab, R. , Becker, C. , Lanz, C. , and Weigel, D. (2015). Genome‐wide analysis of local chromatin packing in *Arabidopsis thaliana* . Genome Res. 25: 246–256. 25367294 10.1101/gr.170332.113 PMC4315298

[jipb70007-bib-0067] Wang, L. , Jia, G. , Jiang, X. , Cao, S. , Chen, Z.J. , and Song, Q. (2021). Altered chromatin architecture and gene expression during polyploidization and domestication of soybean. Plant Cell 33: 1430–1446. 33730165 10.1093/plcell/koab081 PMC8254482

[jipb70007-bib-0068] Wang, Q. , Yung, W.S. , Wang, Z. , and Lam, H.M. (2020). The histone modification H3K4me3 marks functional genes in soybean nodules. Genomics 112: 5282–5294. 32987152 10.1016/j.ygeno.2020.09.052

[jipb70007-bib-0069] Wang, W. , Chandra, A. , Goldman, N. , Yoon, S. , Ferrari, E.K. , Nguyen, S.C. , Joyce, E.F. , and Vahedi, G. (2022). TCF‐1 promotes chromatin interactions across topologically associating domains in T cell progenitors. Nat. Immunol. 23: 1052–1062. 35726060 10.1038/s41590-022-01232-z PMC9728953

[jipb70007-bib-0070] Wang, X. , Teng, B. , He, X. , and Tong, Y. (2013). Classification of glutamine nthetase gene and preliminary functional analysis of the nodule‐predominantly expressed gene GmGS1β2 in soybean. Acta Agronom. Sin. 39: 2145–2153.

[jipb70007-bib-0071] Yan, W. , Chen, D. , Schumacher, J. , Durantini, D. , Engelhorn, J. , Chen, M. , Carles, C.C. , and Kaufmann, K. (2019). Dynamic control of enhancer activity drives stage‐specific gene expression during flower morphogenesis. Nat. Commun. 10: 1705. 30979870 10.1038/s41467-019-09513-2 PMC6461659

[jipb70007-bib-0072] Yang, H. , Luan, Y. , Liu, T. , Lee, H.J. , Fang, L. , Wang, Y. , Wang, X. , Zhang, B. , Jin, Q. , Ang, K.C. , et al. (2020). A map of cis‐regulatory elements and 3D genome structures in zebrafish. Nature 588: 337–343. 33239788 10.1038/s41586-020-2962-9 PMC8183574

[jipb70007-bib-0073] Yang, J. , Gao, F. , and Pan, H. (2025). Essential roles of nodule cysteine‐rich peptides in maintaining the viability of terminally differentiated bacteroids in legume‐rhizobia symbiosis. J. Integr. Plant Biol. 67: 1077–1085. 40105505 10.1111/jipb.13891

[jipb70007-bib-0074] Yang, J. , Lan, L. , Jin, Y. , Yu, N. , Wang, D. , and Wang, E. (2022a). Mechanisms underlying legume‐rhizobium symbioses. J. Integr. Plant Biol. 64: 244–267. 34962095 10.1111/jipb.13207

[jipb70007-bib-0075] Yang, Z. , Du, H. , Xing, X. , Li, W. , Kong, Y. , Li, X. , and Zhang, C. (2022b). A small heat shock protein, GmHSP17.9, from nodule confers symbiotic nitrogen fixation and seed yield in soybean. Plant Biotechnol. J. 20: 103–115. 34487637 10.1111/pbi.13698 PMC8710831

[jipb70007-bib-0076] Yu, G. , Wang, L.G. , Han, Y. , and He, Q.Y. (2012). clusterProfiler: An R package for comparing biological themes among gene clusters. OMICS 16: 284–287. 22455463 10.1089/omi.2011.0118 PMC3339379

[jipb70007-bib-0078] Zhang, Y. , Liu, T. , Meyer, C.A. , Eeckhoute, J. , Johnson, D.S. , Bernstein, B.E. , Nusbaum, C. , Myers, R.M. , Brown, M. , Li, W. , et al. (2008). Model‐based analysis of ChIP‐Seq (MACS). Genome Biol. 9: R137. 18798982 10.1186/gb-2008-9-9-r137 PMC2592715

[jipb70007-bib-0079] Zheng, H. , and Xie, W. (2019). The role of 3D genome organization in development and cell differentiation. Nat. Rev. Mol. Cell Biol. 20: 535–550. 31197269 10.1038/s41580-019-0132-4

[jipb70007-bib-0080] Zhu, B. , Zhang, W. , Zhang, T. , Liu, B. , and Jiang, J. (2015). Genome‐wide prediction and validation of intergenic enhancers in Arabidopsis using open chromatin signatures. Plant Cell 27: 2415–2426. 26373455 10.1105/tpc.15.00537 PMC4815101

