# Supplementary material for: Dynamic 3D chromatin organization and epigenetic regulation of gene expression in peanut nodules
Source: J Integr Plant Biol. 2025 Aug 13;67(10):2624–42. doi: 10.1111/jipb.70007 (PMC12498064; doi:10.1111/jipb.70007)
Supplement: Supplementary file 1 — Figure S1. Three‐dimensional model of whole chromosomes in peanut roots and nodules Figure S2. Single‐chromosome interactions in peanut roots and nodules at a 400‐kb resolution Figure S3. Length distribution of genomic compartments Figure S4. Distribution of gene count and GC content in GCs Figure S5. Functional annotation of genes associated with Compartment A/B transitions Figure S6. Correlation analysis of ATAC‐seq data Figure S7. Characterization of OCRs and epigenome markers in peanut roots Figure S8. Screening of differentially expressed genes Figure S9. GO enrichment results for DEGs from the Nodule versus Root comparison Figure S10. KEGG enrichment results for DEGs from the Nodule versus Root comparison Figure S11. Number of root‐ and nodule‐enriched genes associated with different chromatin features that are differentially expressed between roots and nodules Figure S12. Nodule‐enriched differentially expressed genes and chromatin states in roots and nodules Figure S13. KEGG enrichment analysis of genes located in the TAD boundary of the nodules Figure S14. KEGG enrichment analysis of genes located in the insulation region of the nodules Figure S15. Association study of TADs border region and with epigenetic modifications Figure S16. Example of differentially presented loops around the ahNIN gene that was highly expressed in root nodules Figure S17. Analysis of the chromatin loop around the ahNIN Figure S18. Diagram of the genomic information of the differentially presented loop around the AhMsrA gene that is highly expressed in root nodules Figure S19. Phenotypic analysis of AhMsrA‐RNA interference in peanut nodulation Figure S20. Gene editing status of AhMsrA knockout roots Figure S21. Schematic diagram of enhancer identification in peanut roots and root nodules Figure S22. Enrichment analysis of interaction genes with gain enhancers in nodule Hi‐C compared with root Hi‐C Figure S23. OCRs and nodule‐enriched genes associated with nodule‐specific enhancers [file JIPB-67-2624-s001.docx]

**Supplemental data**


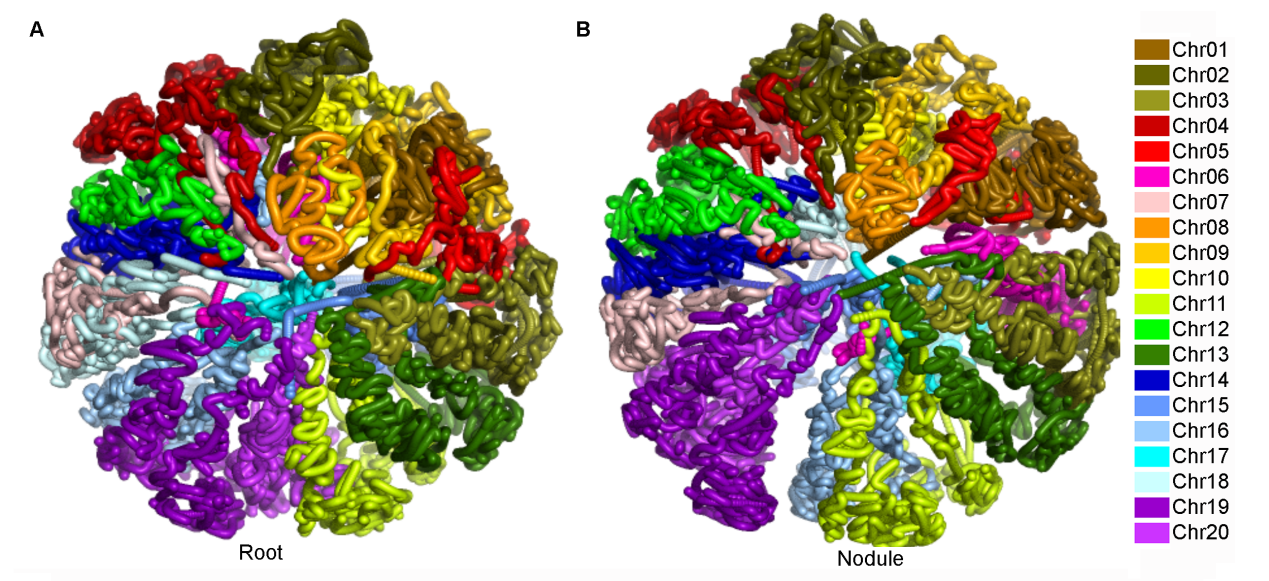


**Figure S1. Three-dimensional model of whole chromosomes in peanut roots and nodules.** Each color represents one chromosome.


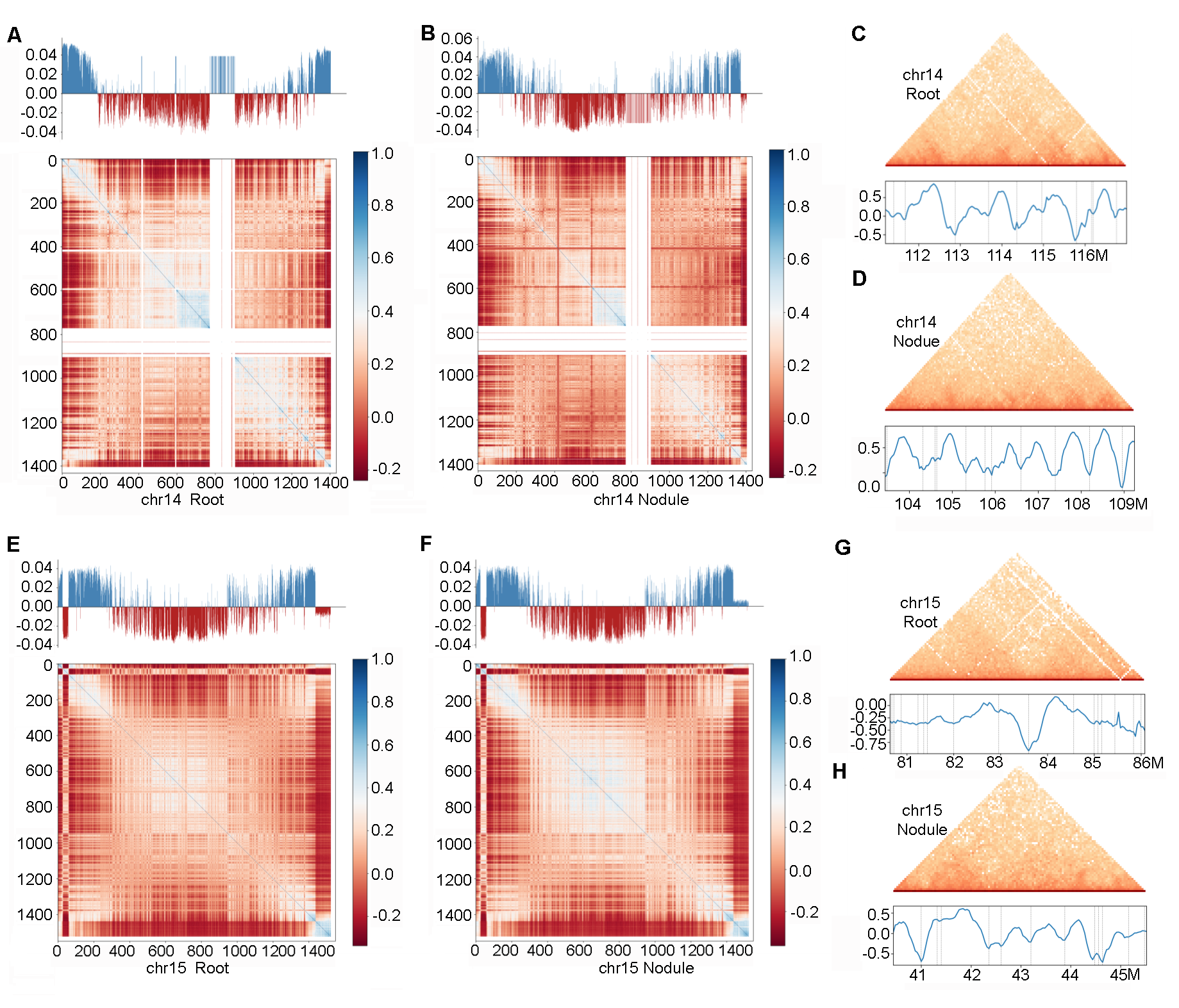


**Figure S2. Single chromosome interactions in peanut roots and nodules at a 400-kb resolution.** Single chromosomal interactions of chr14 in peanut roots and nodules at a 400-kb resolution (A–D). The upper track shows compartments A (blue histogram) and B (red histogram). The bottom track shows chromatin interaction represented by chr14 (A,B). E. Each triangle distributed diagonally is represented as a topologically associated domain (TAD) in peanut roots and nodules (C, D). Single chromosomal interactions of chr15 in peanut roots and nodules at a 400-kb resolution (E–H). The upper track shows compartments A (blue histogram) and B (red histogram). The bottom track shows chromatin interaction represented by chr14 (E, F). E. Each triangle distributed diagonally is represented as a TAD in peanut roots and nodules (G, H).


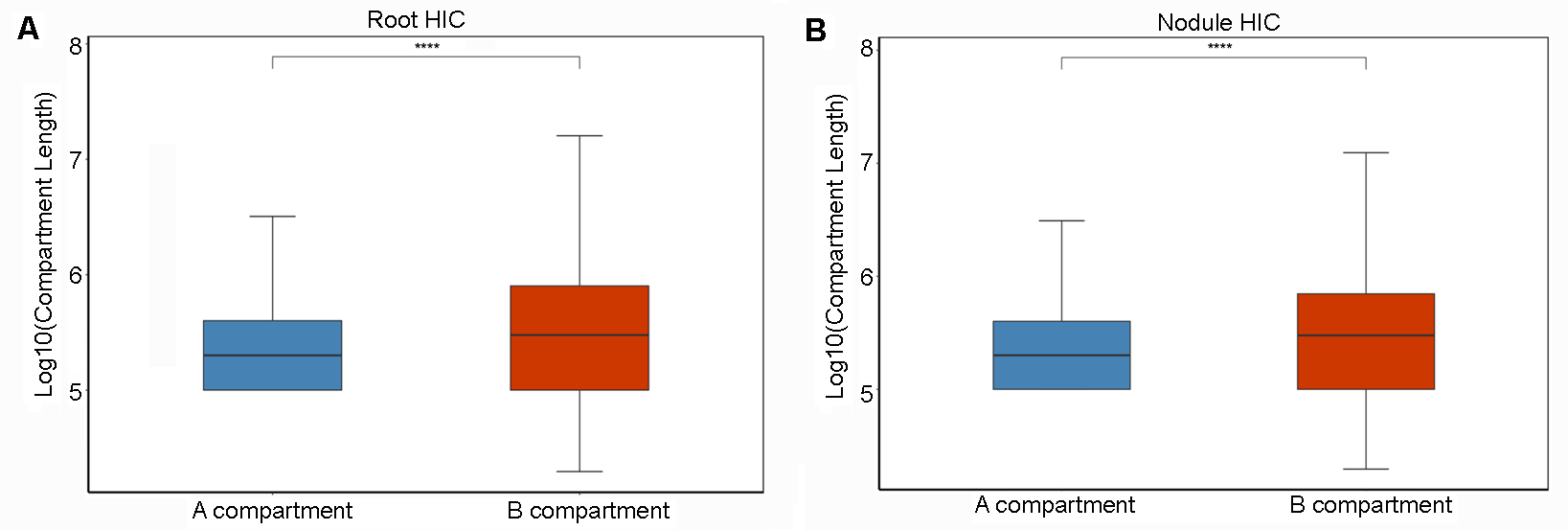


**Figure S3. Length distribution of genomic compartments.** Based on the results of compartment analysis, the contiguous bins of compartments A and B were merged to quantify the length distribution of both compartments across the entire genome of the roots (A) and nodules (B).


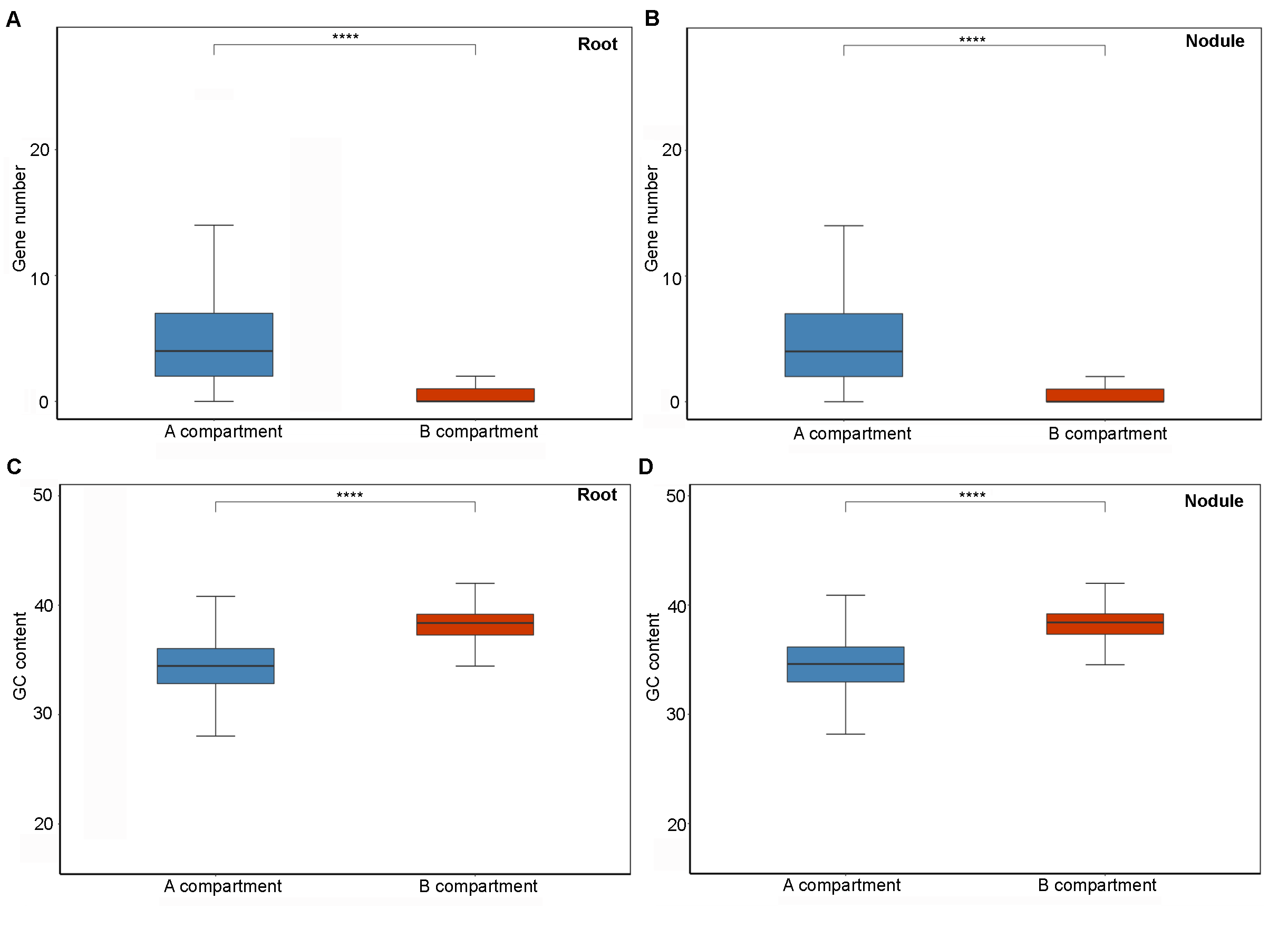


**Figure S4. Distribution of gene count and GC content in genomic compartments.** Based on the results of compartment analysis, the number of genes (A, C) and GC content (B, D) within each bin were determined in compartments A and B of the whole genome of the roots (A, B) and nodules (C, D).


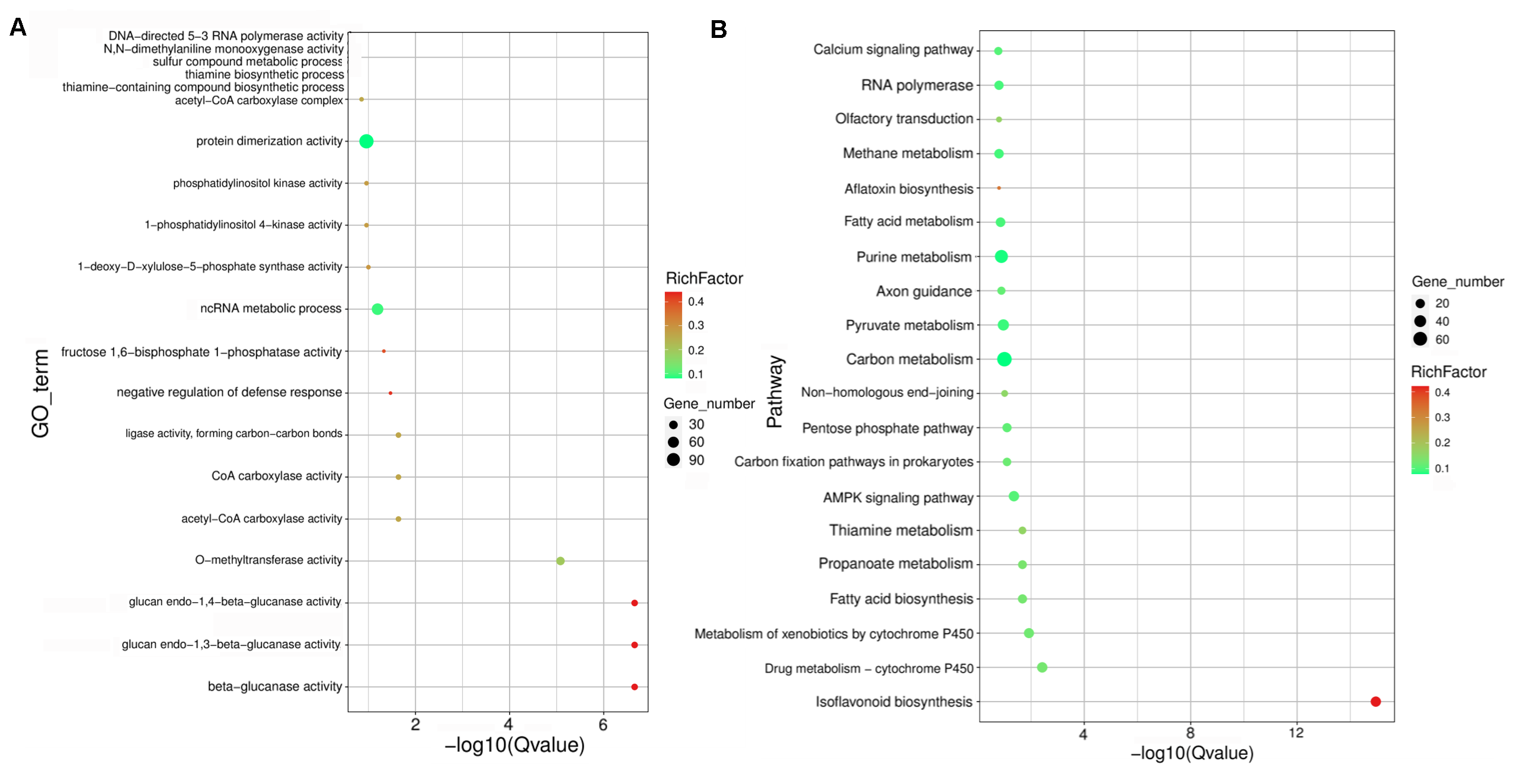


**Figure S5. Functional annotation of genes associated with compartment A/B transitions.** GO enrichment results for genes that underwent compartment A/B transitions (A), and KEGG enrichment results for these genes (B).


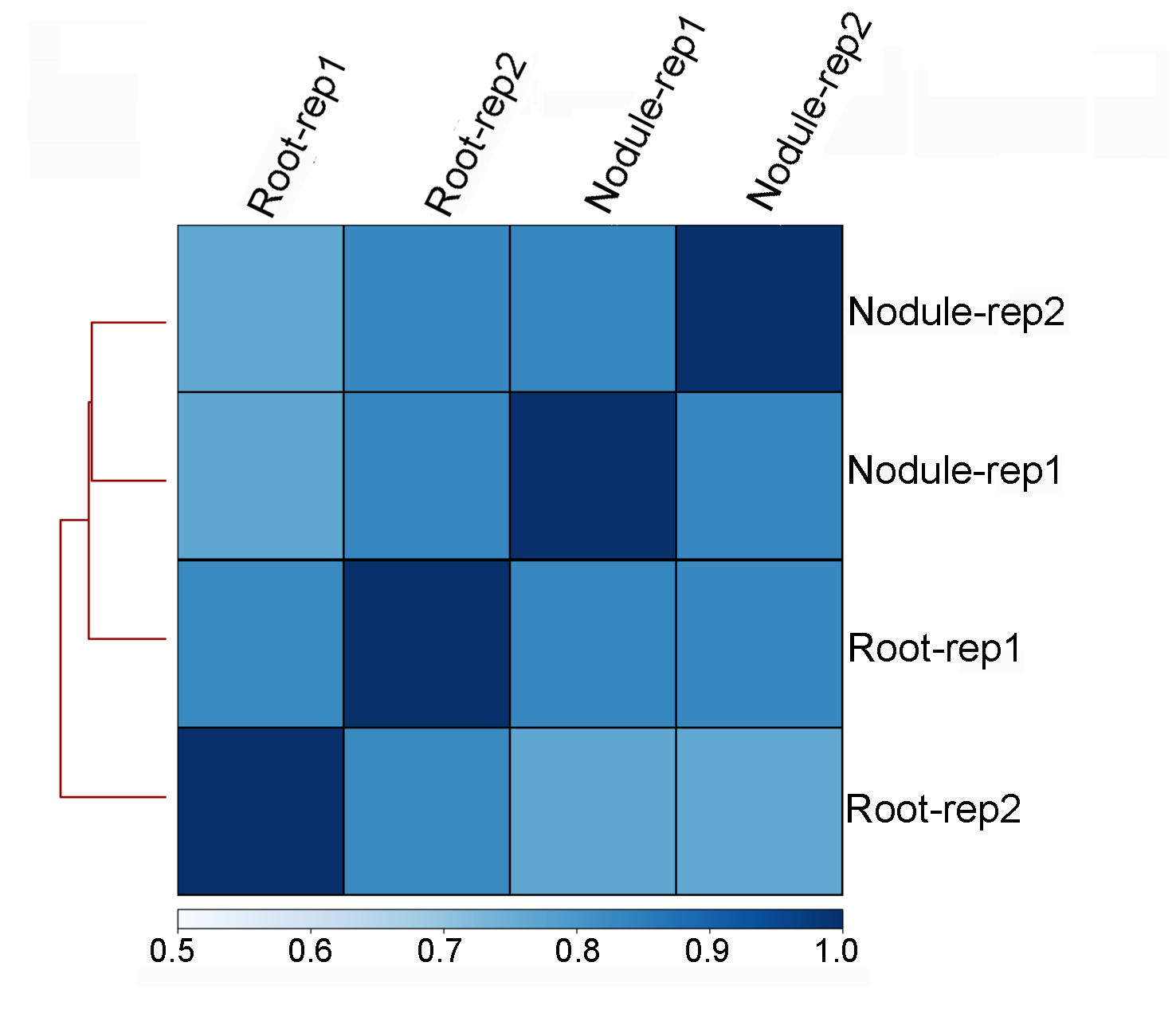


**Figure S6. Correlation analysis of ATAC-seq data.**

**
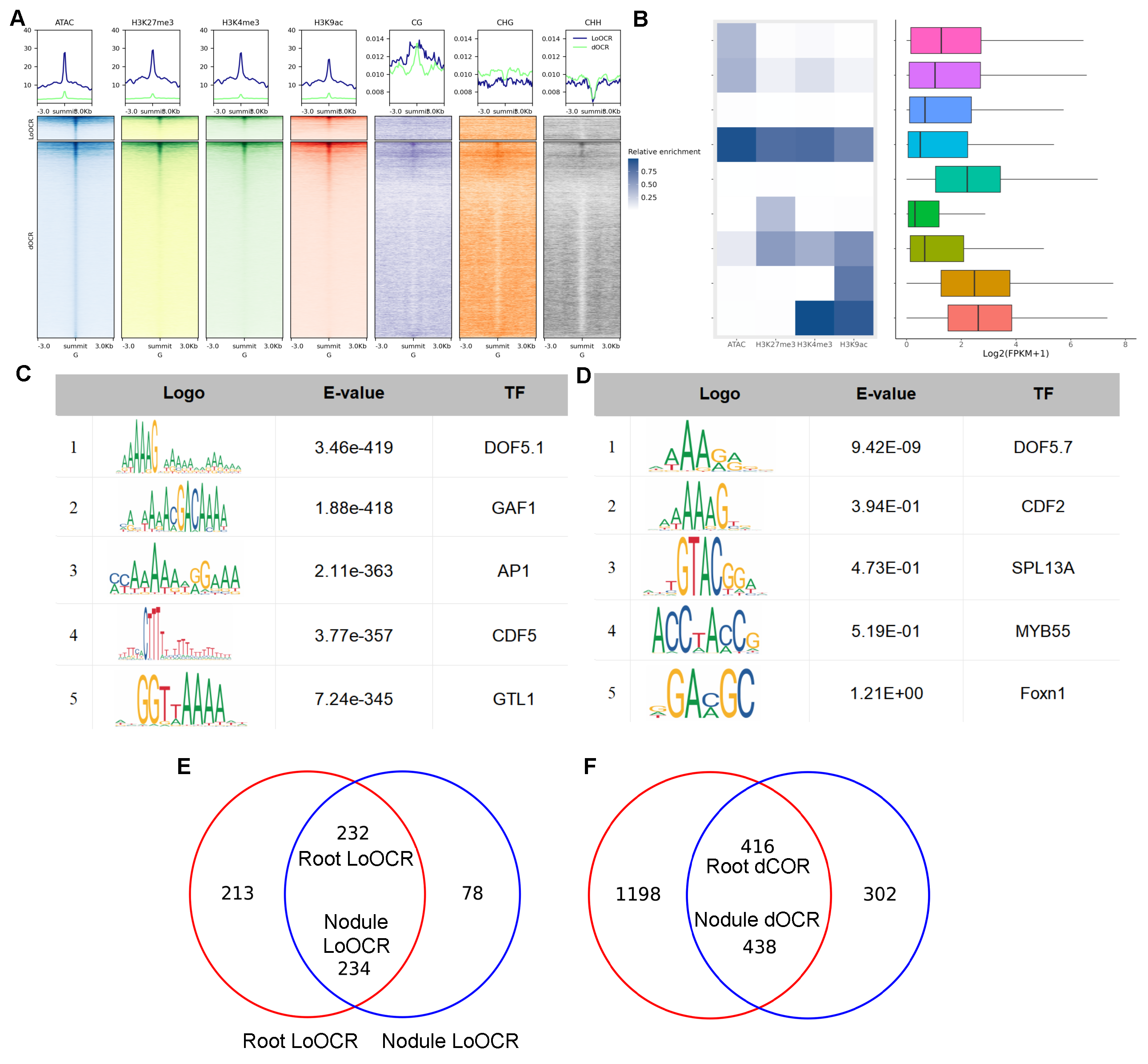
**

**Figure S7. Characterization of OCRs and epigenome markers in peanut roots.** A. Chromatin accessibility (ATAC-seq), and H3K4me3, H3K9ac, H3K27me3, and DNA methylation (CG) profiles on a selected region of chromosome 13 (8860–8940 kb). Vertical dotted lines show an example of dynamic chromatin accessibility and histone modifications with the altered transcription of a nearby gene between roots and nodules. B. Correlation between gene expression and different clusters of chromatin accessibility and histone modifications in peanut roots. C, D. DNA motifs enriched in LoOCRs (C), and dOCRs (D) of roots. The corresponding top 5 candidate motif-binding TFs are shown. E, F. Number of root and nodule specific NREs containing LoOCRs (E) and dOCRs (F).


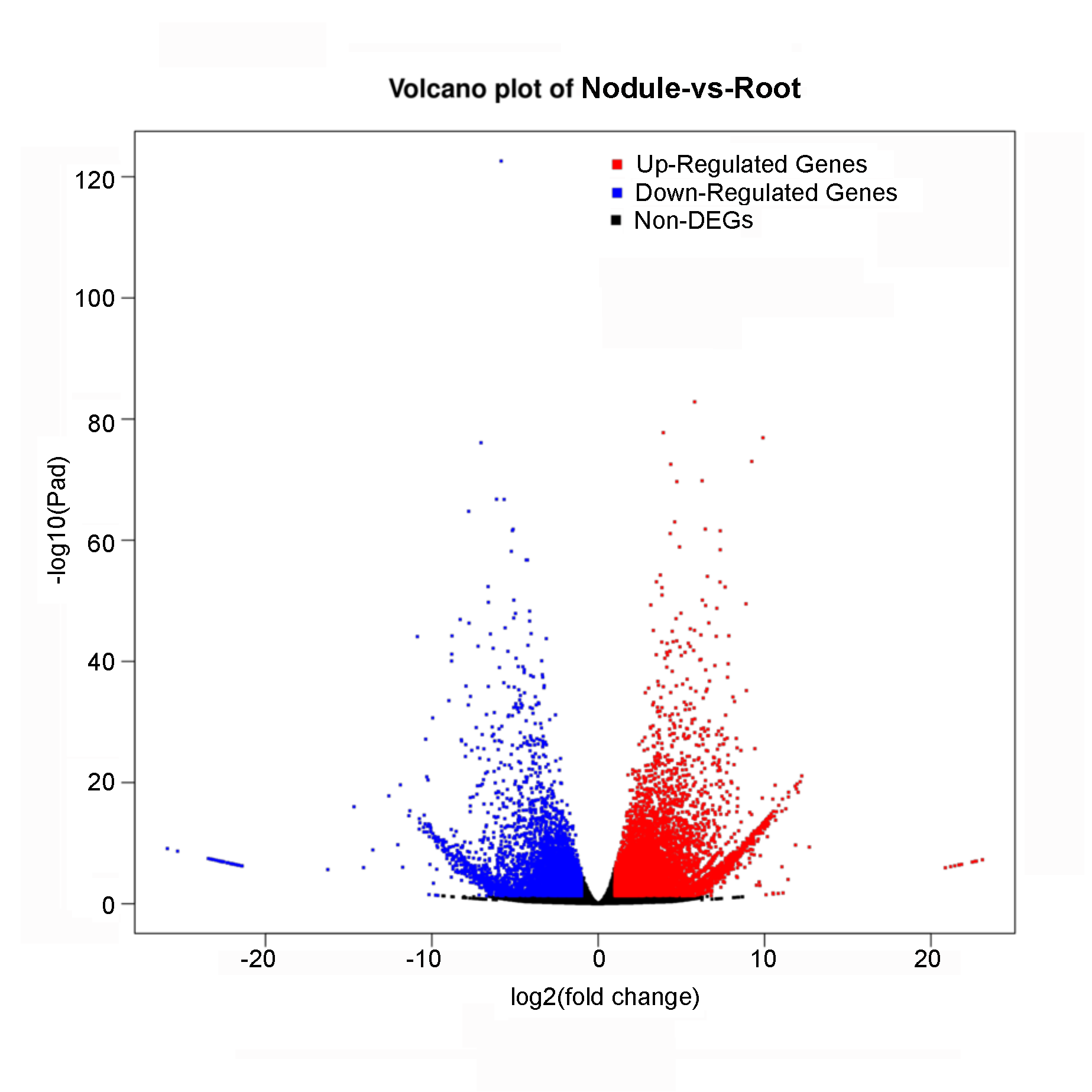


**Figure S8. Screening of differentially expressed genes.** To visually display the distribution of FDR and fold change (FC) values for all genes between the two groups of samples, volcano plots were generated for each group comparison.


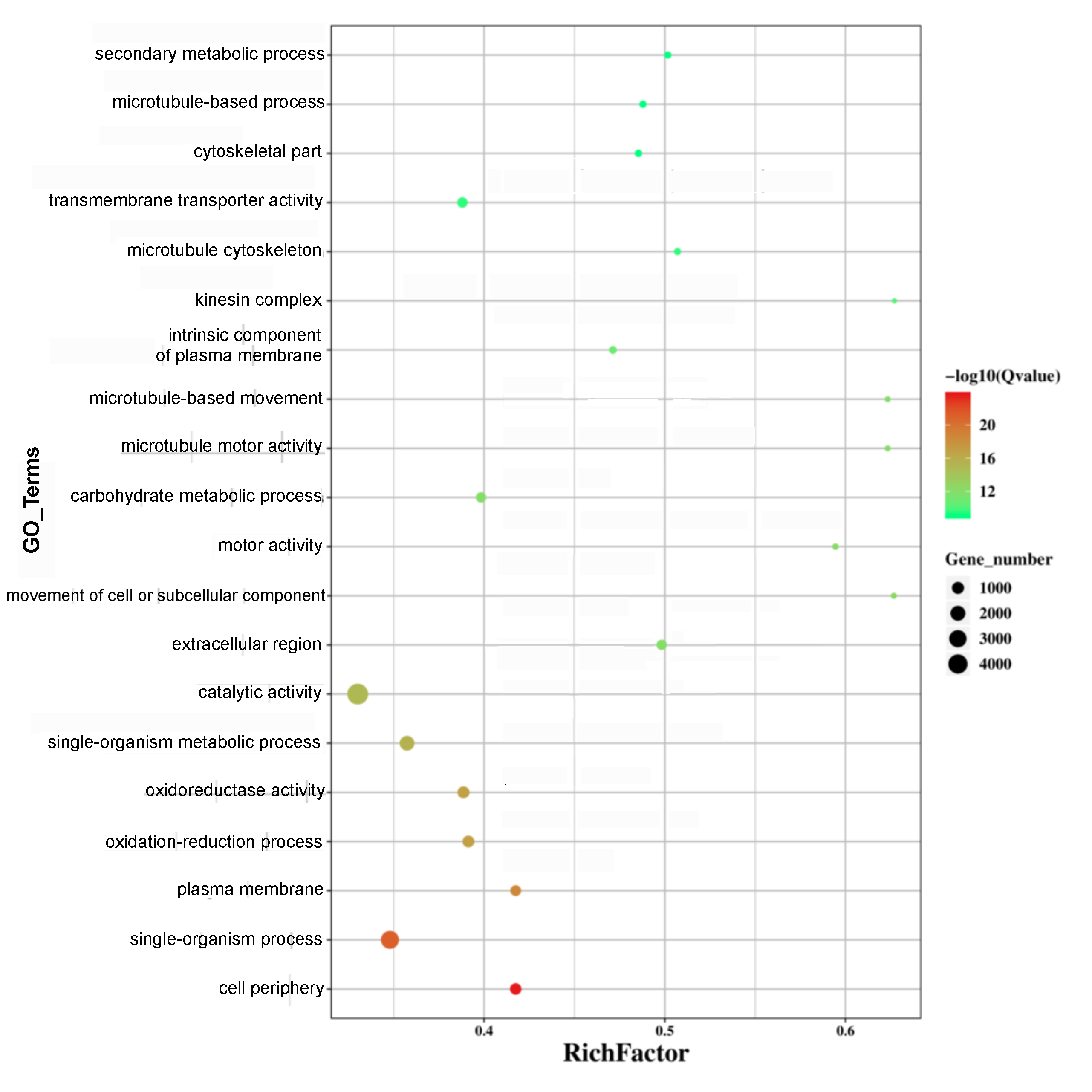
**Figure S9. GO enrichment results for DEGs from the Nodule vs Root comparison.**


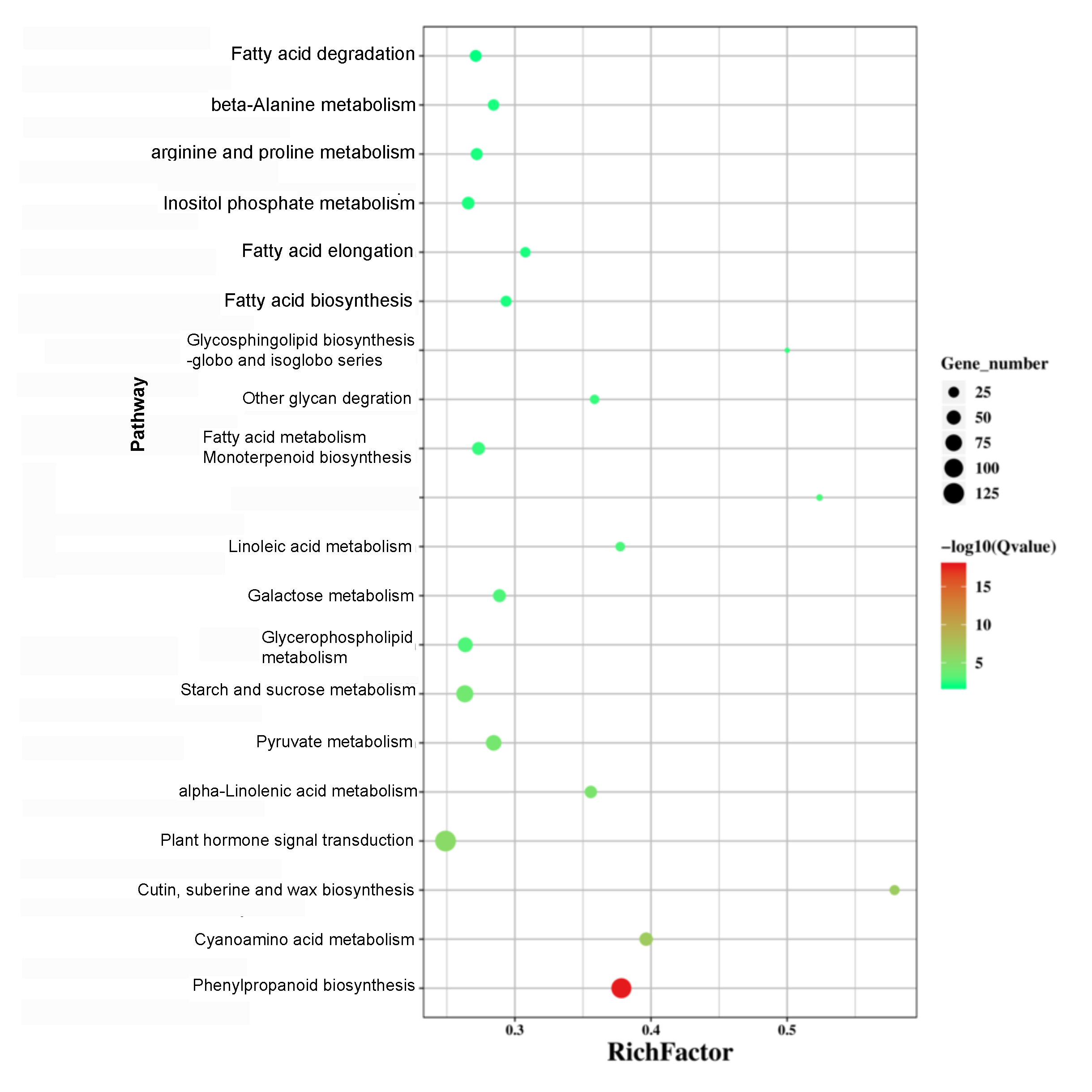


**Figure S10. KEGG enrichment results for DEGs from the Nodule vs Root comparison.**


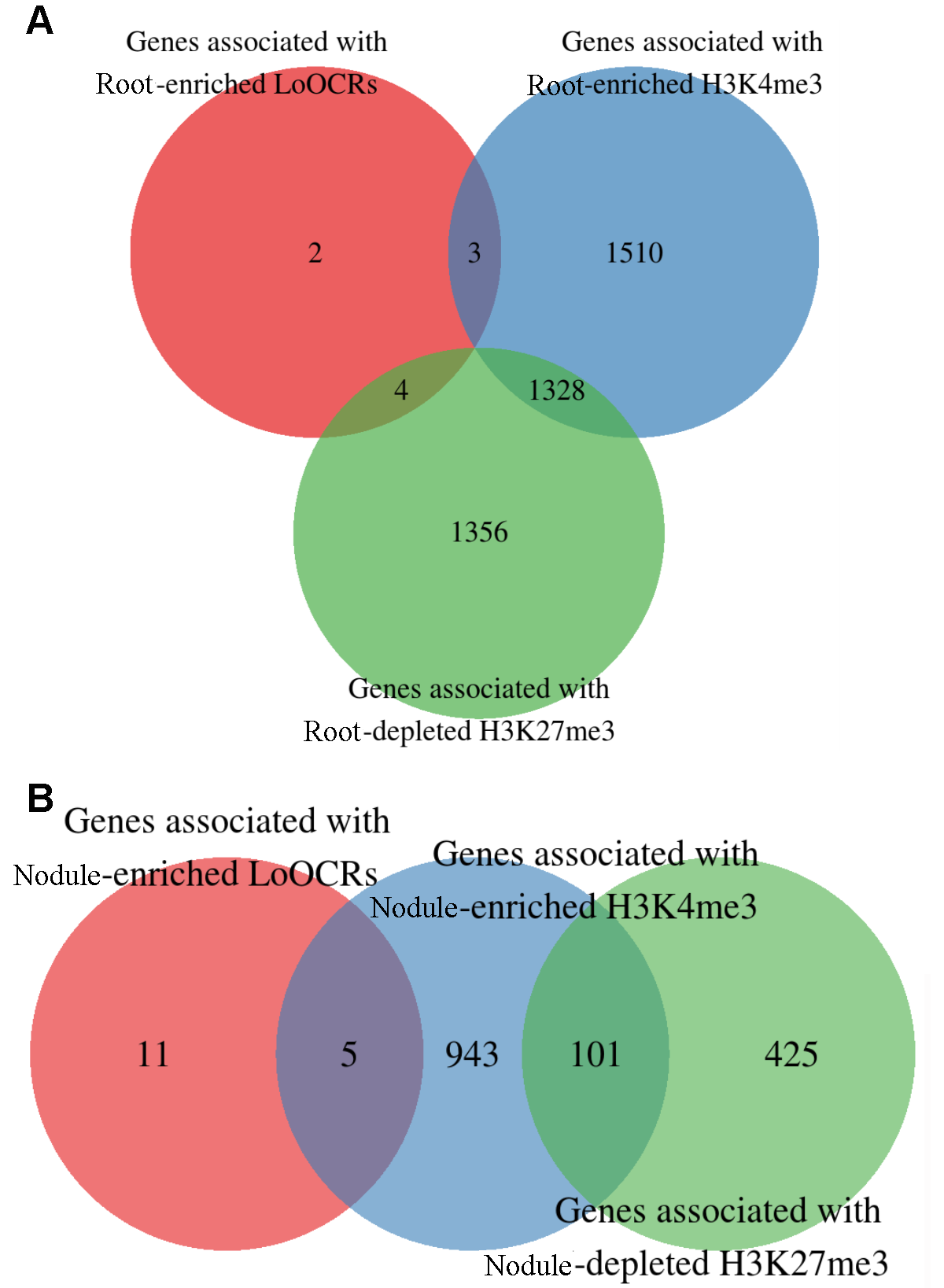


**Figure S11. Number of root- (A) and nodule-enriched (B) genes associated with different chromatin features that are differentially expressed between roots and nodules.**


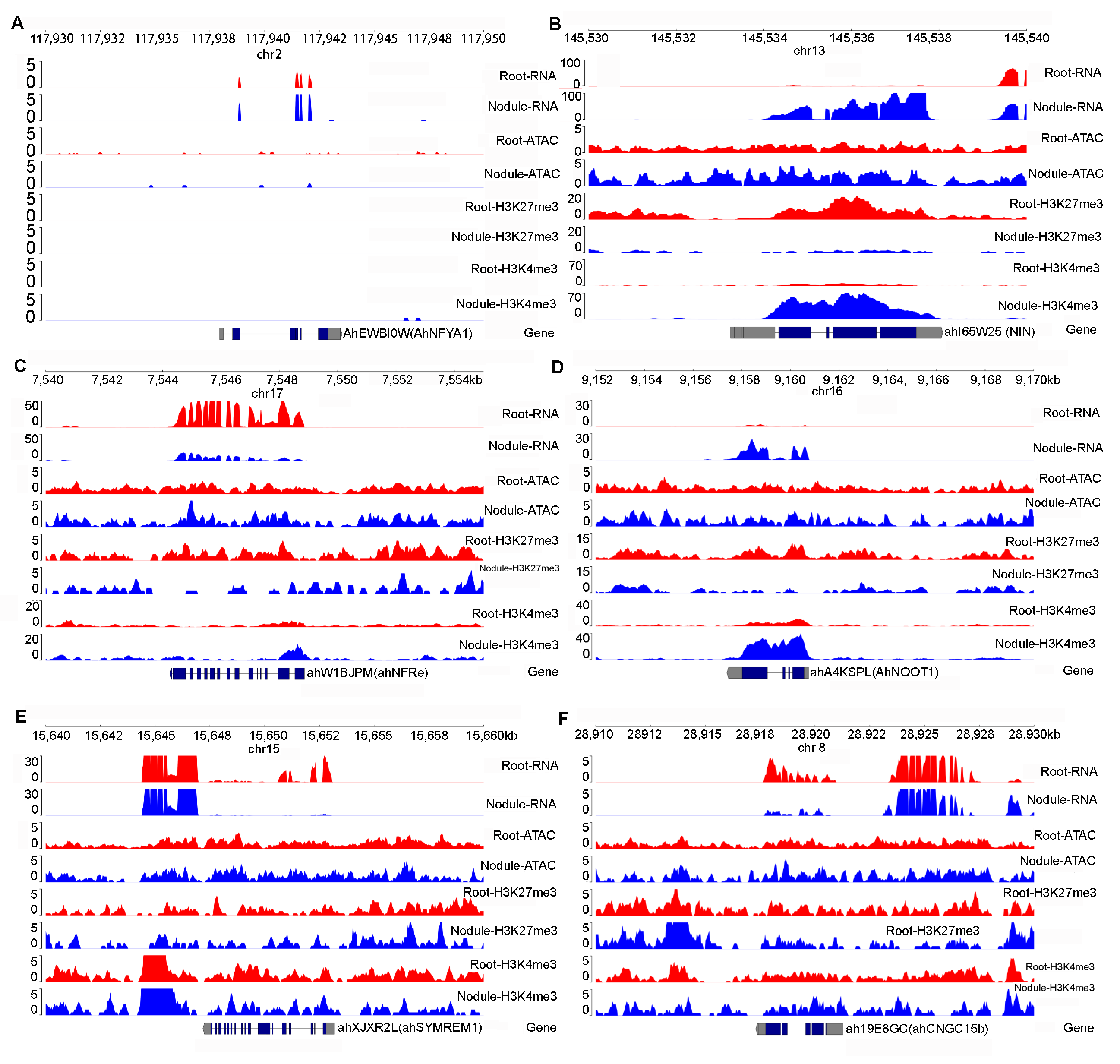


**Figure S12. Nodule-enriched differentially expressed genes and chromatin states in roots and nodules.** *ahNFYA1* (A), *ahNINa* (B), *ahNFRe* (C), *ahNOOT1* (D), *ahSYMREM1* (E), and *ahCNGC15b* (F).


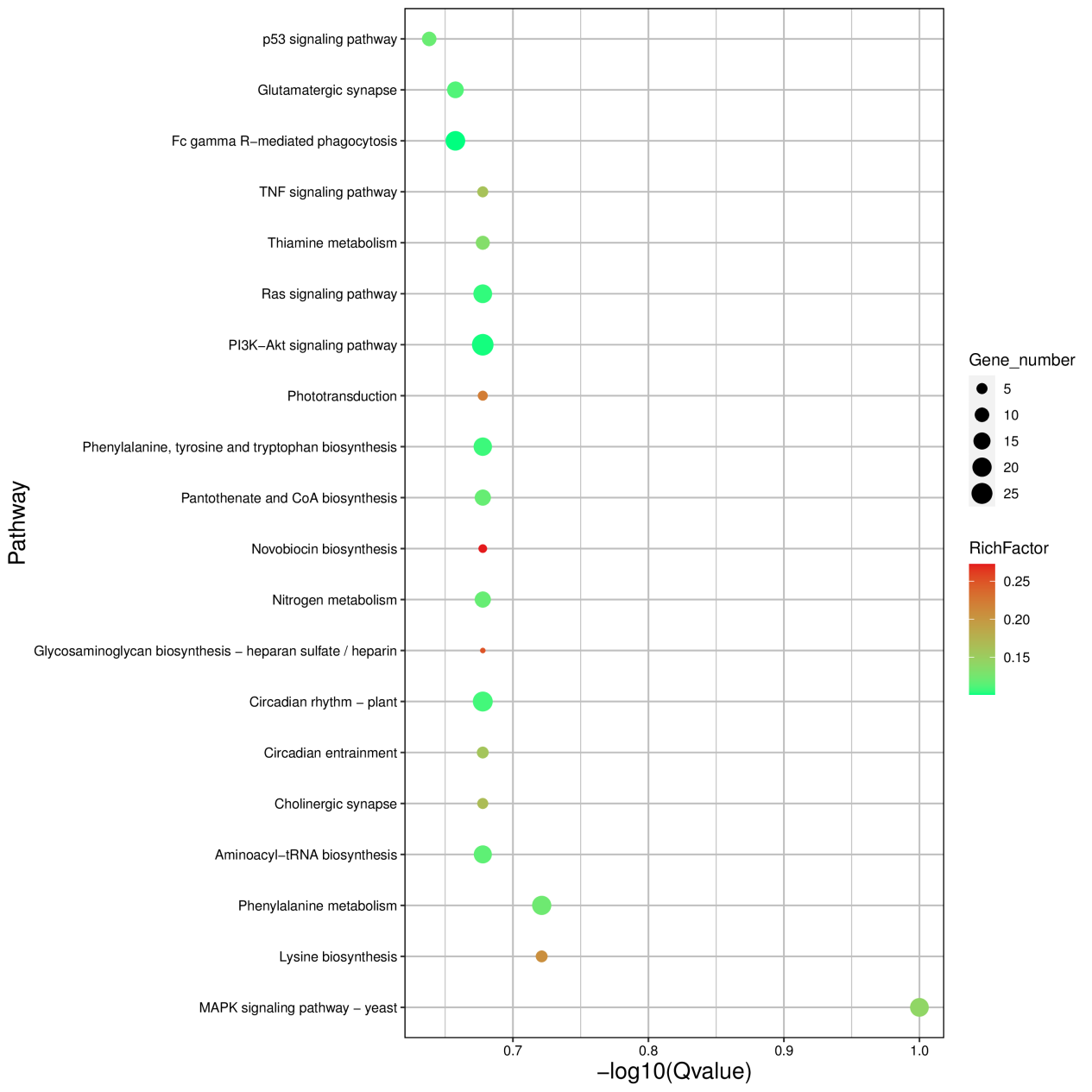


**Figure S13. KEGG enrichment analysis of genes located in the TAD boundary of the nodules.**


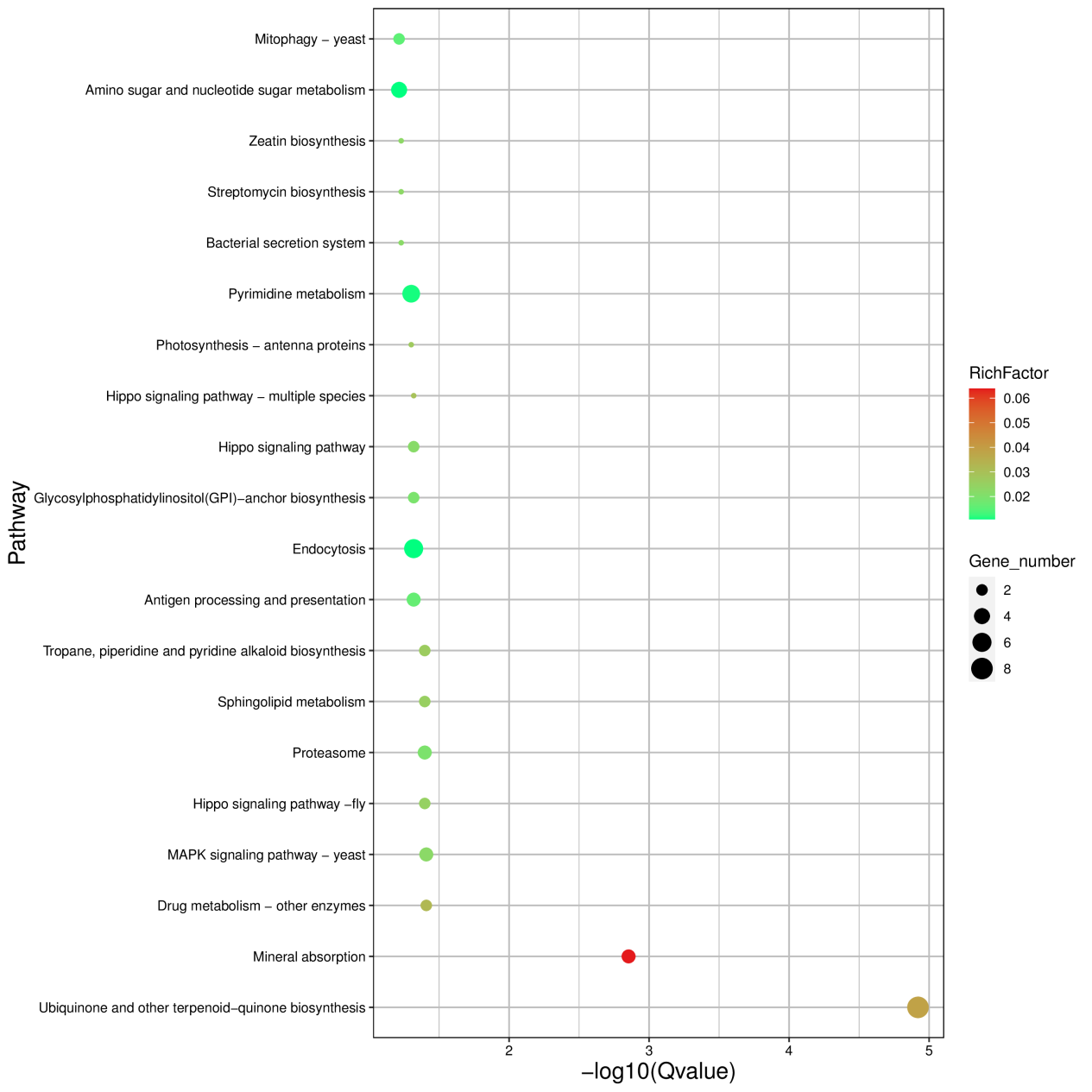


**Figure S14. KEGG enrichment analysis of genes located in the insulation region of the nodules.**


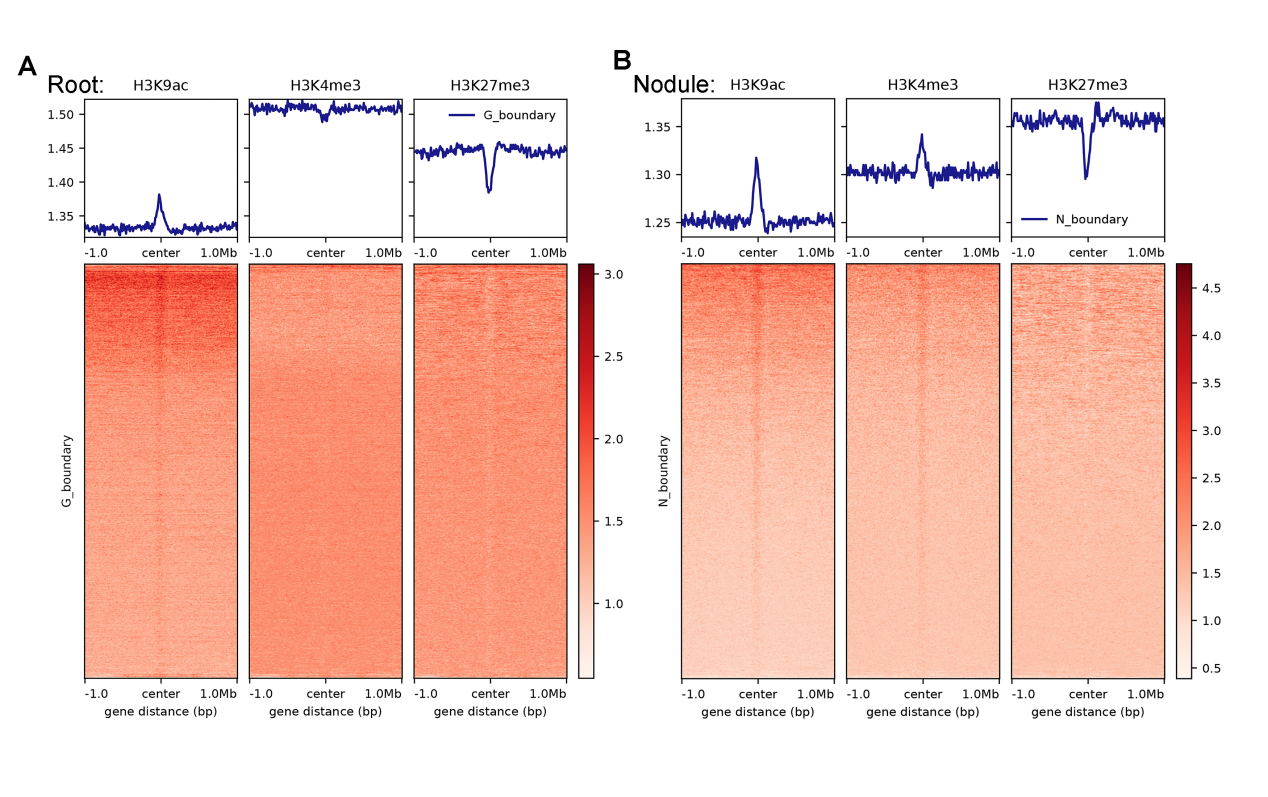


**Figure S15. Association study of TADs border region and with epigenetic modifications.**


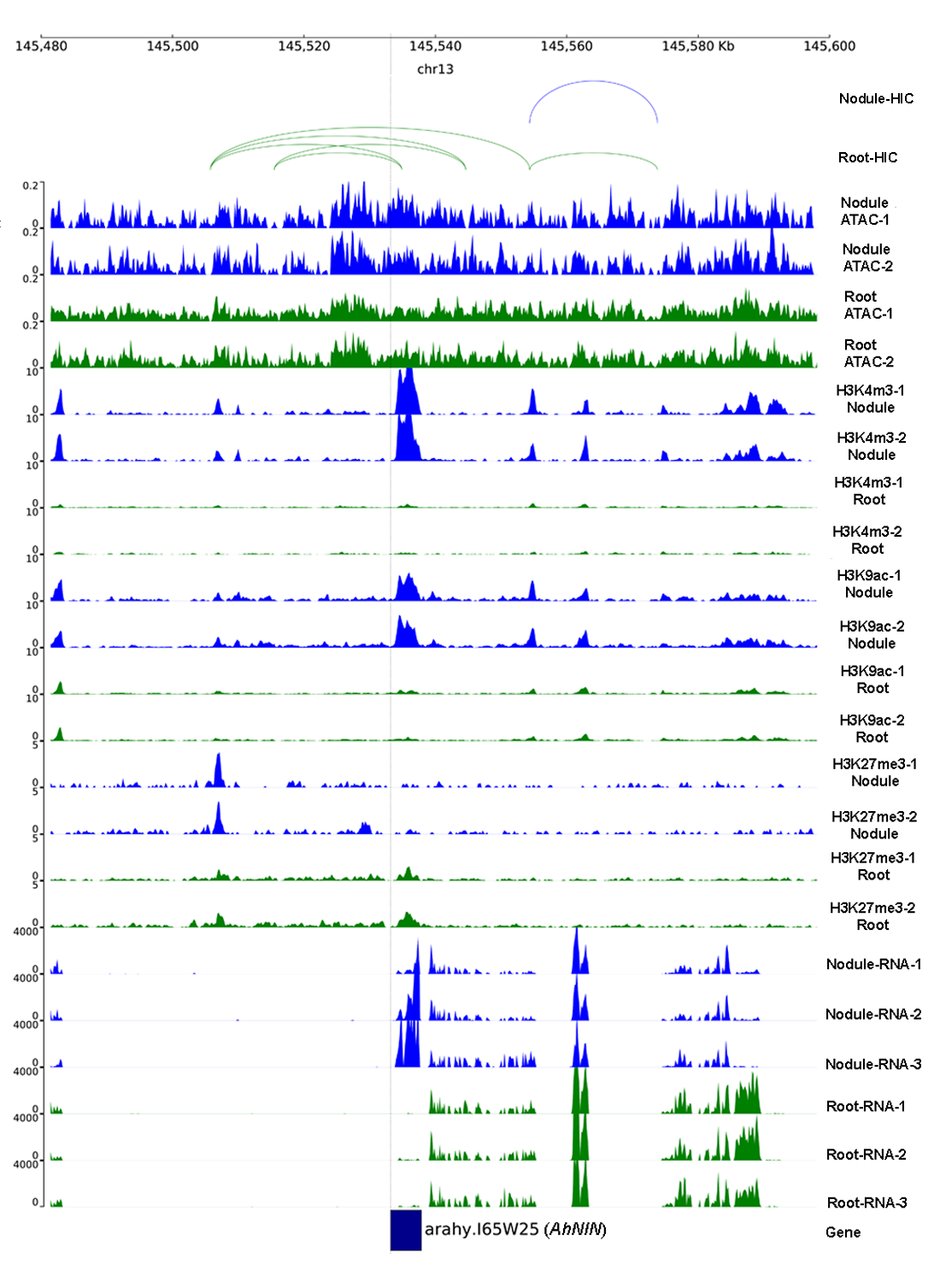


**Figure S16. Example of a differentially presented loops around the *ahNIN* gene that was highly expressed in root nodules.**


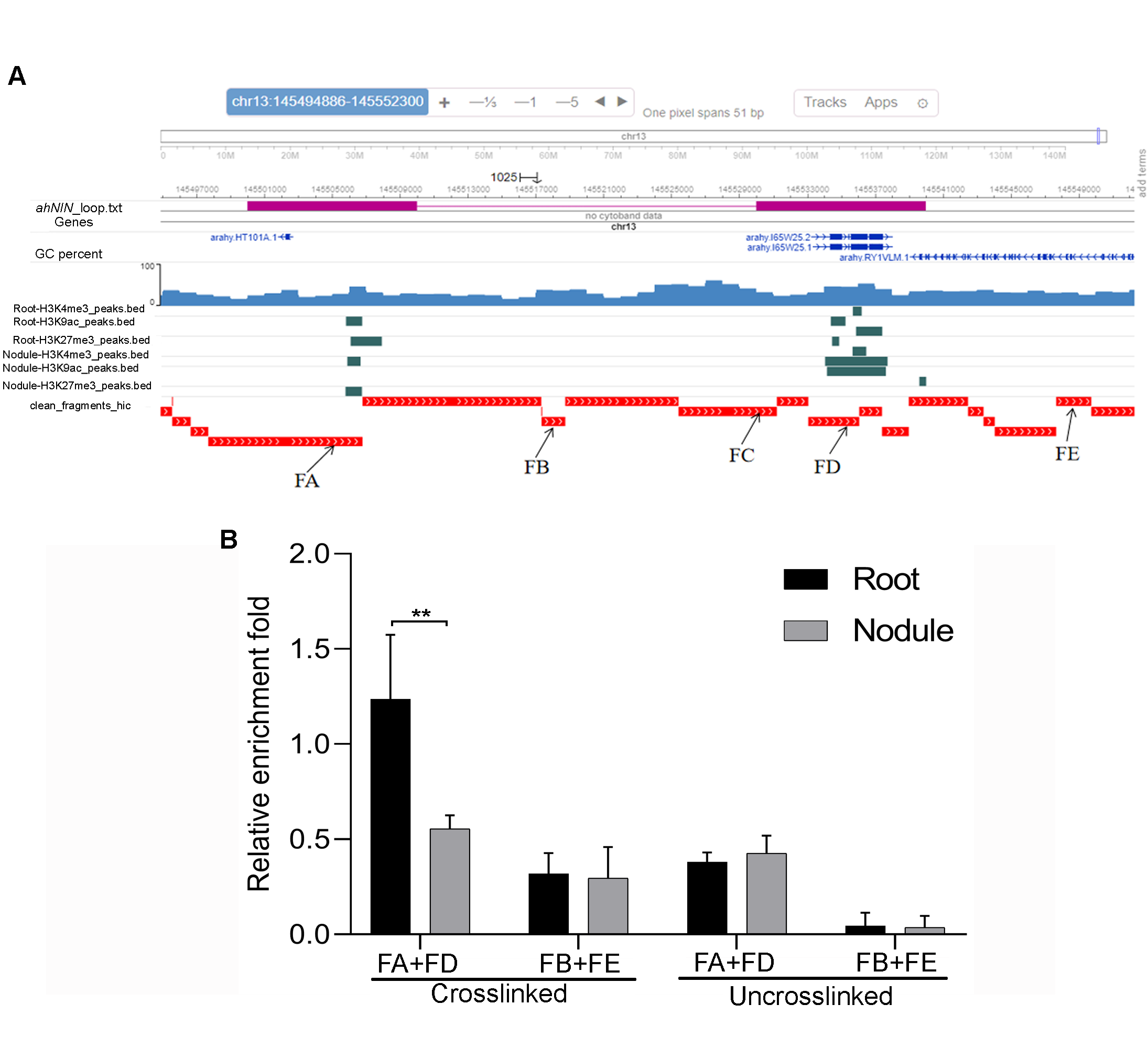


**Figure S17. Analysis of the chromatin loop around the *ahNIN*.** Diagram of the genomic profile in peanut roots and nodules, including loop information, GC content, H3K4me3, H3K9ac, and H3K27me3. *Hind* III digested fragments were used to design the 3C-qPCR primers. FA and FD primers were used to examine the chromatin loop (marked with red boxes) (A). 3C-qPCR examining the chromatin loop in roots and nodules. Crosslinked and non-crosslinked samples were analyzed with the anchor primer set (FA+FD), and PCR with the primer set (FB+FE) served as a low interaction frequency control. Error bars represent SDs from three biological repeats. *** P* < 0.01 (B).


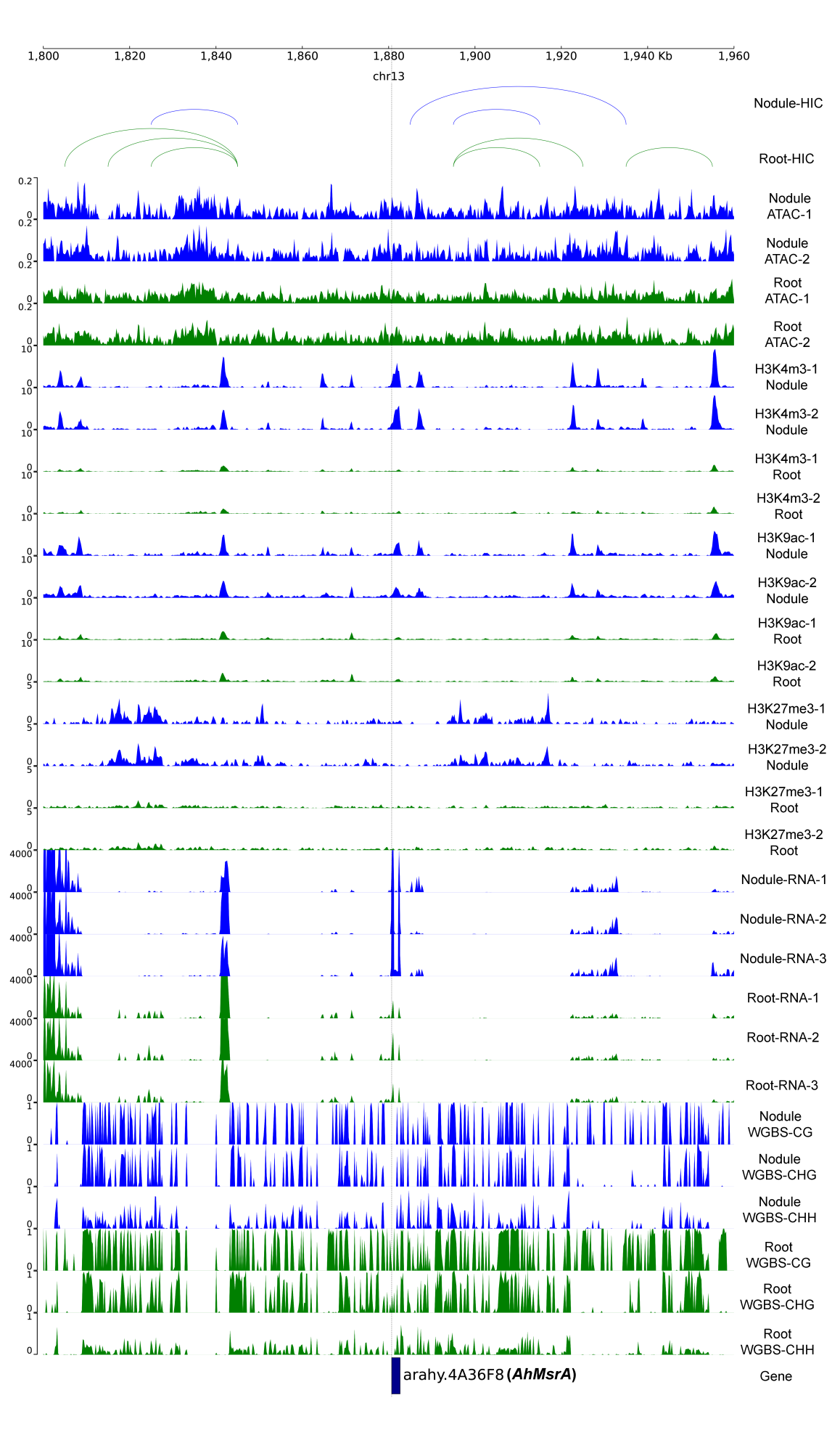


**Figure S18. Diagram of the genomic information of the differentially presented loop around the *AhMsrA* gene that is highly expressed in root nodules.**


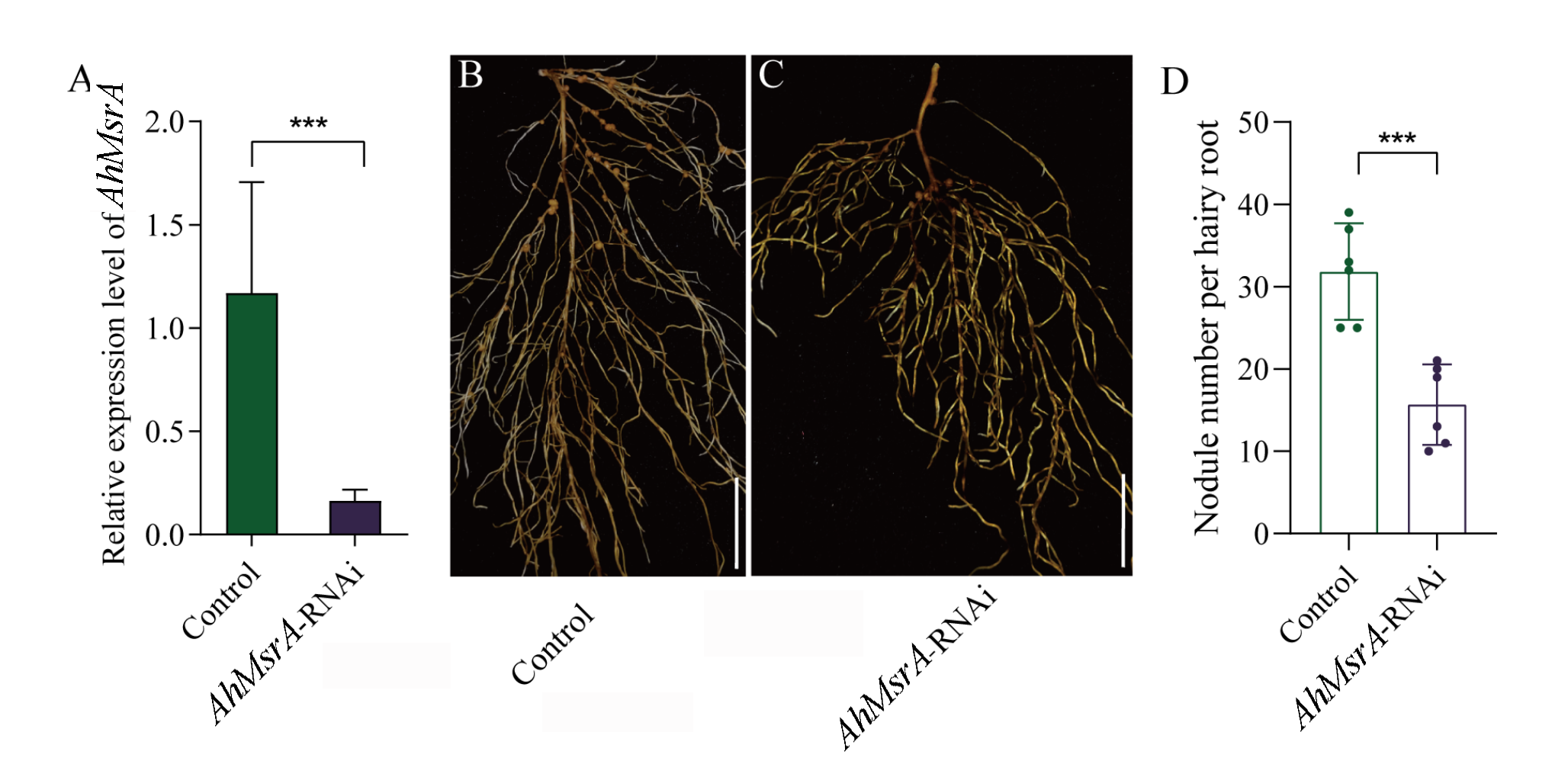


**Figure S19. Phenotypic analysis of *AhMsrA*-RNAi in peanut nodulation.** Expression level of transgenic hairy roots harboring the empty vector and 35S:*AhMsrA* (A). The expression levels were normalized against the housekeeping gene of *AhActin*. Student’s t-test was performed (****p* < 0.001, n = 15). D. Nodulation of transgenic roots expressing EV1 (B) and 35S:*AhMsrA* (C) at 28 DAI. Bar = 1 cm. Quantitative statistics of nodule number per hairy root carrying EV and 35S:*AhMsrA* at 28 DAI. Values are the mean ± SD. For each biological replicate, 12 hairy roots were collected (n = 12, Student’s t-test; ****p* < 0.001) (D).


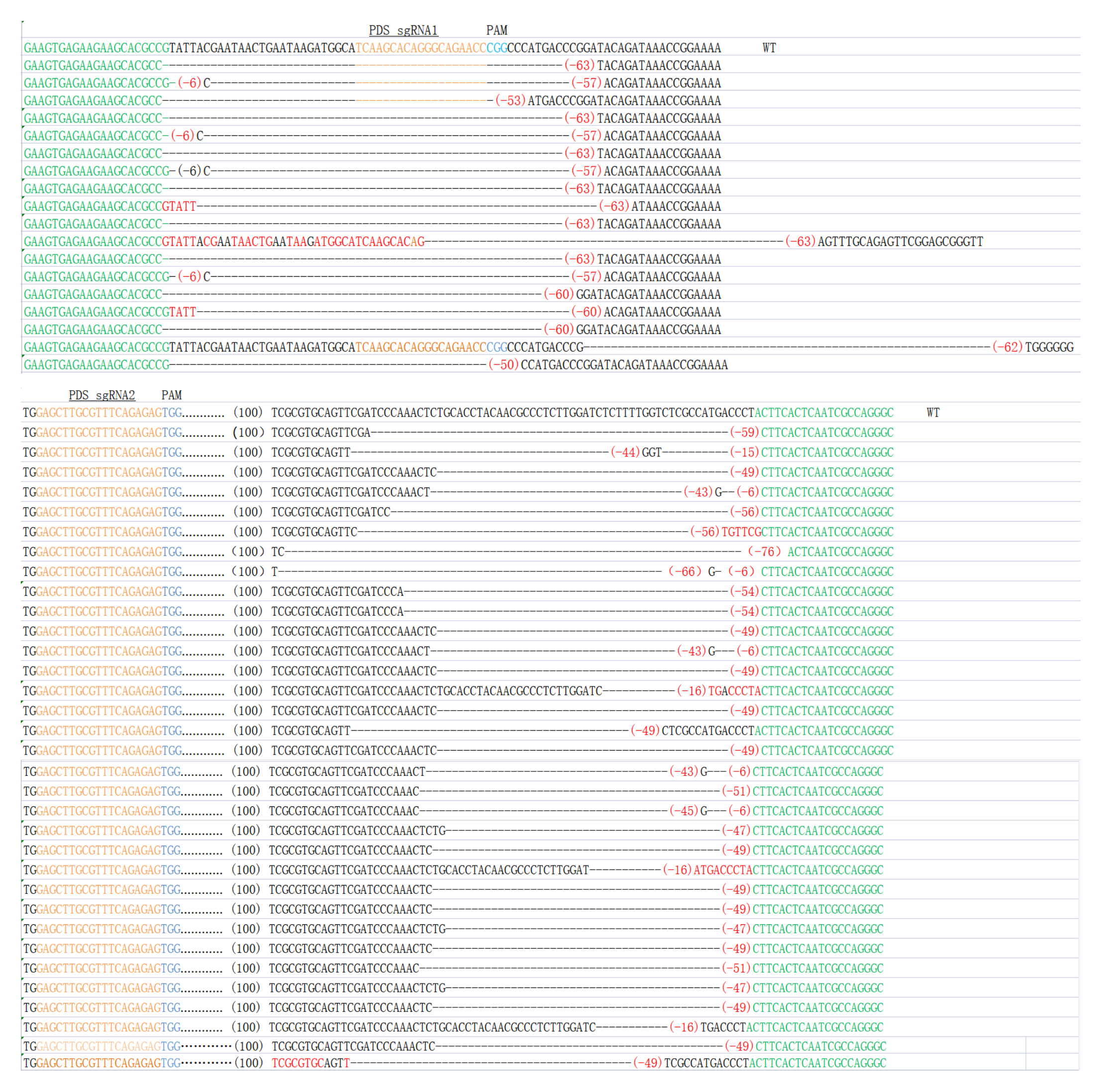


**Figure S20. Gene editing status of *AhMsrA* knockout roots.**


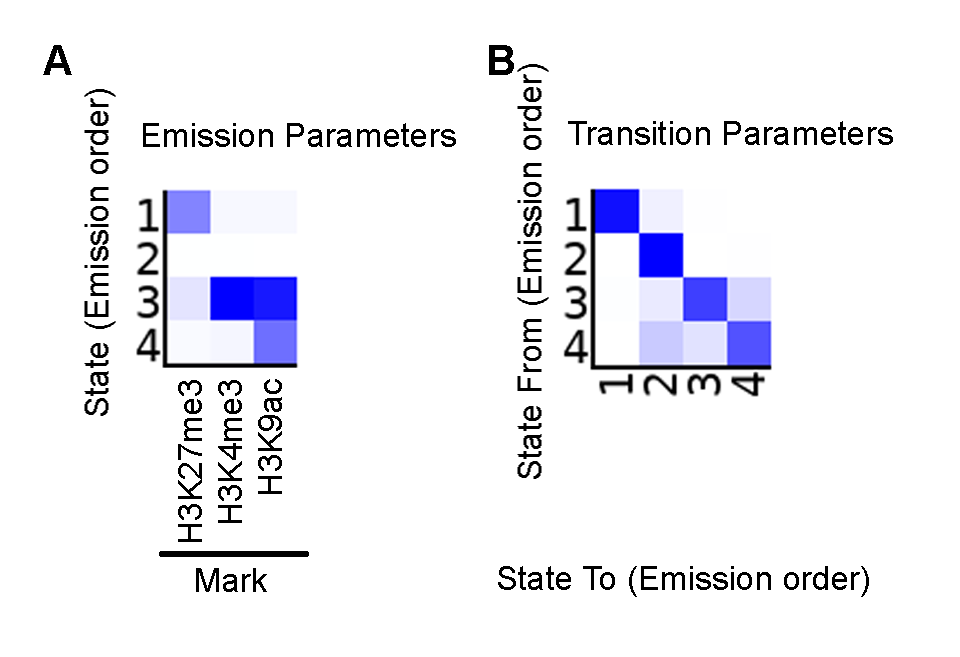


**Figure S21. Schematic diagram of enhancer identification in peanut roots and root nodules.**


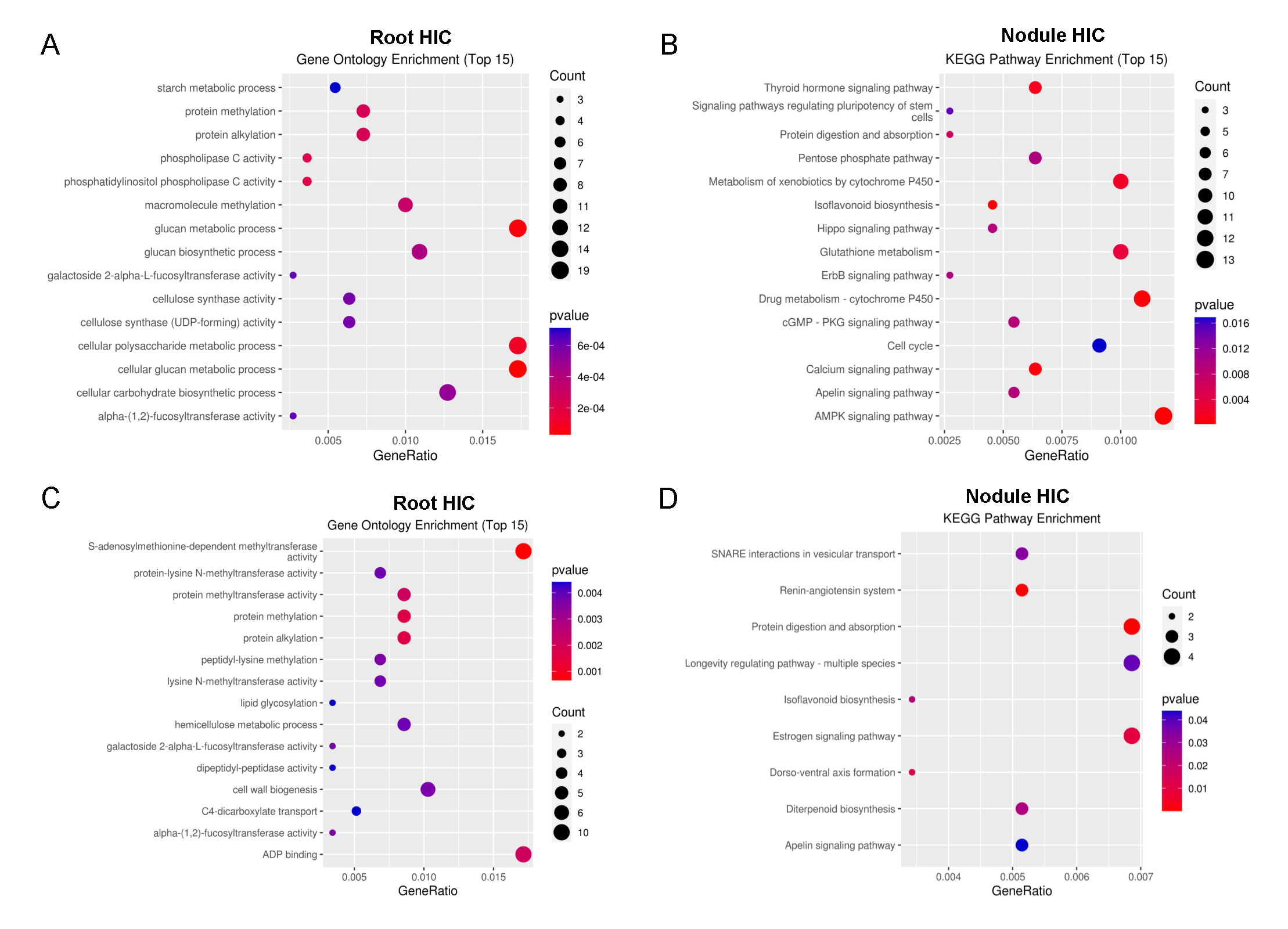


**Figure S22. Enrichment analysis of interaction genes with gain enhancers in nodule HIC compared with root HIC.** KEGG enrichment results for these DEGs from the Nodule vs Root comparison. GO enrichment analysis results of interaction genes with gain enhancers in peanut roots (A). KEGG enrichment analysis results of interaction genes with gain enhancers in peanut roots (B). GO enrichment analysis results of interaction genes with gain enhancers in peanut nodules (C). KEGG enrichment analysis results of interaction genes with gain enhancers in peanut nodules (D).


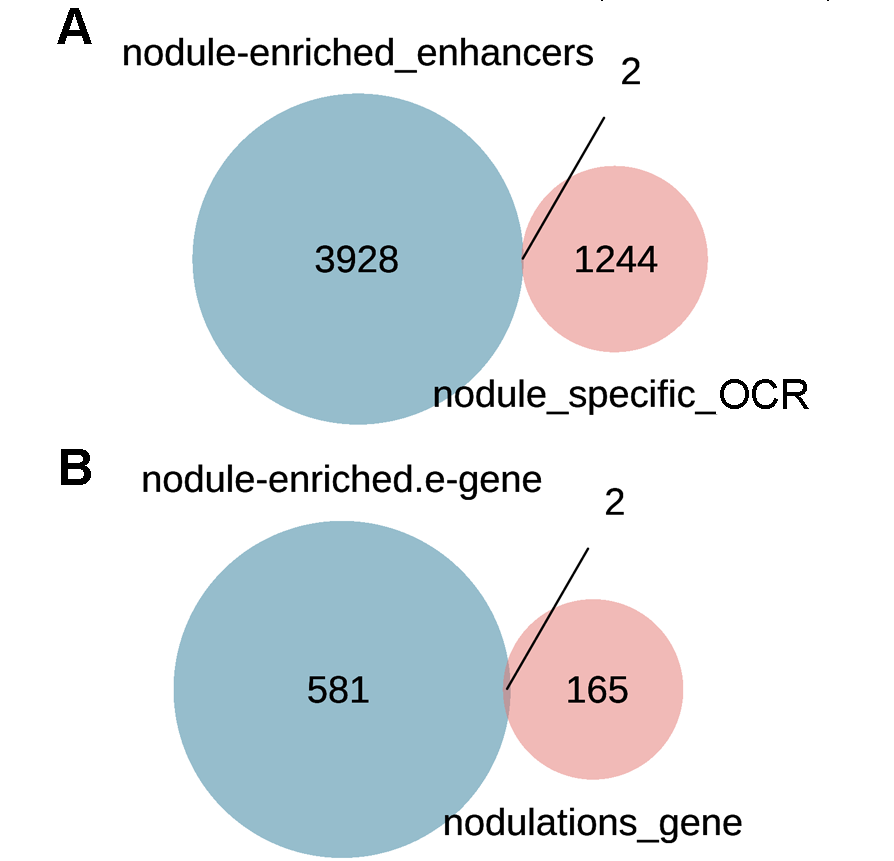


**Figure S23. OCRs (A) and nodule-enriched genes (B) associated with nodule specific enhancers.**
